# Genetic Analysis and Detection of *fliC*_H1_ and *fliC*_H12_ Genes Coding for Serologically Closely Related Flagellar Antigens in Human and Animal Pathogenic *Escherichia coli*

**DOI:** 10.3389/fmicb.2016.00135

**Published:** 2016-02-15

**Authors:** Lothar Beutin, Sabine Delannoy, Patrick Fach

**Affiliations:** ^1^Department of Biology, Chemistry, Pharmacy, Institute for Biology - Microbiology, Freie Universität BerlinBerlin, Germany; ^2^Université Paris-Est, Anses, Food Safety Laboratory, IdentyPathMaisons-Alfort, France

**Keywords:** *E. coli*, molecular serotyping, fliC type H1 gene, fliC type H12 gene, STEC, ExPEC

## Abstract

The *E. coli* flagellar types H1 and H12 show a high serological cross-reactivity and molecular serotyping appears an advantageous method to establish a clear discrimination between these flagellar types. Analysis of *fliC*_H1_ and *fliC*_H12_ gene sequences showed that they were 97.5% identical at the nucleotide level. Because of this high degree of homology we developed a two-step real-time PCR detection procedure for reliable discrimination of H1 and H12 flagellar types in *E. coli*. In the first step, a real-time PCR assay for common detection of both *fliC*_H1_ and *fliC*_H12_ genes is used, followed in a second step by real-time PCR assays for specific detection of *fliC*_H1_ and *fliC*_H12_, respectively. The real-time PCR for common detection of *fliC*_H1_ and *fliC*_H12_ demonstrated 100% sensitivity and specificity as it reacted with all tested *E. coli* H1 and H12 strains and not with any of the reference strains encoding all the other 51 flagellar antigens. The *fliC*_H1_ and *fliC*_H12_ gene specific assays detected all *E. coli* H1 and all *E. coli* H12 strains, respectively (100% sensitivity). However, both assays showed cross-reactions with some flagellar type reference strains different from H1 and H12. The real-time PCR assays developed in this study can be used in combination for the detection and identification of *E. coli* H1 and H12 strains isolated from different sources.

## Introduction

Strains belonging to the species of *Escherichia coli* are ubiquitous as commensals in the gut of humans and warm-blooded animals. Apart from their role as beneficial microbes, some *E. coli* strains are known to behave as human and animal pathogens, causing a wide spectrum of extraintestinal and enteric diseases, with urinary tract infection and diarrhea as most frequent (Kaper et al., 2004; Stenutz et al., 2006). Pathogenic and apathogenic *E. coli* cannot be discerned from each other by their morphology, cultural properties or fermentation reactions. As a consequence, serotyping is used since the 1940s as a diagnostic tool for identification of animal and human pathogenic *E. coli* strains (Orskov and Orskov, 1984).

*E. coli* serogroups are commonly defined by the antigenic properties of the lipopolysaccharide which is part of the outer membrane (O-antigen) (Stenutz et al., 2006). Motile *E. coli* strains can be additionally typed for their flagellar filaments (H-antigen) (Orskov and Orskov, 1984). *E. coli* O- and H-antisera are usually produced by immunization of rabbits with respective reference strains (Orskov and Orskov, 1984; Edwards and Ewing, 1986). At present, 182 O-antigens and 53 H-antigens have been described (Scheutz et al., 2004; Scheutz and Strockbine, 2005). The resulting O:H serotype (for example O157:H7) is commonly used for describing *E. coli* isolates (Bettelheim, 1978; Orskov and Orskov, 1984).

Complete serotyping of *E. coli* is laborious and time-consuming and performed only in a few specialized reference laboratories worldwide. Moreover, cross-reactivity which is observed between some *E. coli* O-groups and H-types can complicate the interpretation of serotyping results. Last but not least, serotyping fails if autoagglutinating (O-antigen or H-antigen rough) and non-motile (NM) *E. coli* strains have to be examined (Orskov and Orskov, 1984; Edwards and Ewing, 1986). For these reasons, attempts were made to substitute serotyping by molecular typing of O-antigen and H-antigen encoding genes.

In the recent years, the nucleotide sequences of all known O and H-antigen genes in *E. coli* have been elucidated (Wang et al., 2003; Iguchi et al., 2015a). Molecular methods such as PCR and nucleotide sequencing have been successfully employed for typing of O- and H-antigen genes in *E. coli* (Beutin and Fach, 2014; Joensen et al., 2015; Iguchi et al., 2015b). Molecular serotyping was shown to be specific and sensitive and can substitute conventional serological detection of *E. coli* surface antigens (Bugarel et al., 2010; Fratamico et al., 2011; Clotilde et al., 2015; Iguchi et al., 2015b; Joensen et al., 2015). In contrast to serotyping, molecular detection of O- and H-antigen genes is easier and faster to perform and O-rough and non-motile strains can be typed on the basis of their O- and H-antigen genes (Beutin and Fach, 2014; Joensen et al., 2015).

We have previously investigated the genetic variability of flagellar types H19, H25 and H28 in *E. coli* (Beutin et al., 2015a,b). These flagellar types are widespread in strains belonging to numerous O-serogroups but are also associated with enterohemorrhagic *E. coli* O145:H25, O145:H28, and O121:H19 strains. By nucleotide sequence analysis of *fli*C (flagellin) genes encoding H19, H25, and H28 flagella we have observed a high genetic variability among *fliC*_H19_, *flic*_H25_, and *fliC*_H28_ alleles, respectively. To some part, this sequence alterations were associated with some O-groups of strains which allowed the development of real-time PCR protocols for specific typing of flagellar variants encoded by enterohemorrhagic *E. coli* O145:H25, O145:H28, and O121:H19 strains (Beutin et al., 2015a,b). Such real-time PCR protocols were found useful for improvement of horizontal real-time PCR detection methods for EHEC from food samples (Beutin et al., 2015a,b).

In this work, we compared *E. coli fliC* genes that encode flagellar types H1 and H12. These flagellar types show a high serological cross-reactivity and cross-absorbed H1 and H12 antisera are used for definite H-typing (Orskov and Orskov, 1984; Edwards and Ewing, 1986). Moreover, three subtypes of H1 were detected by serological typing using factor specific antisera (Ratiner et al., 1995). Serological cross reactions may cause confounding results in diagnostic laboratories where absorbed antisera are not available. The development of molecular typing procedures for reliable detection of H1 and H12 flagellar types could overcome this specific problem.

A clear discrimination between *E. coli* flagellar types H1 and H12 has a value for clinical diagnostics and for epidemiological investigations. Some human isolates of Shiga Toxin-producing *E. coli* (STEC) express H1 or H12 flagella (Scheutz and Strockbine, 2005). Moreover, flagellar type H1 is clinically significant as it is associated with worldwide occurring extraintestinal pathogenic *E. coli* (ExPEC) strains carrying capsular polysaccharides (O2:K2:H1, O4:K12:H1, O6:K2:H1, O6:K5:H1, O7:K1:H1, O15:K52:H1) that cause cystitis, pyelonephritis and urosepsis (Orskov and Orskov, 1985; Johnson et al., 1994, 2005, 2006; Olesen et al., 2009). Adherent-invasive *E. coli* (AIEC) O83:H1 strains were associated with Crohn's disease in human patients (Allen et al., 2008; Nash et al., 2010) and flagellar type H1 is associated with biofilm formation and invasive properties of AIEC strains (Eaves-Pyles et al., 2008; Martinez-Medina et al., 2009) as well as with intestinal colonization (Martinez-Medina and Garcia-Gil, 2014). Moreover, H1-type flagellum is a characteristic trait of Shiga toxin 2e-producing *E. coli* O139:H1 strains which are a major cause of edema disease in pigs (Tschape et al., 1992; Frydendahl, 2002; Fairbrother et al., 2005; Beutin et al., 2008). Conversely, the flagellar type H12 has not been associated with pathogenic *E. coli*, except from human enterotoxigenic O78:H12 and O128:H12 strains (Orskov and Orskov, 1977; Echeverria et al., 1982; Shaheen et al., 2004).

In this work we have analyzed the nucleotide sequences of *E. coli* H1 and H12 strains in order to detect characteristic *fliC* sequence alterations corresponding with these closely related H-types. Subsequently, we have developed a real-time PCR procedure for reliable discrimination of H1 and H12 flagellar types in *E. coli*. The protocol should be useful for diagnostic and epidemiological investigations of human and animal pathogenic strains of *E. coli*.

## Materials and methods

### Bacteria

*E. coli* strains used in this study were derived from the collections of the National Reference Laboratory for *E. coli* (NRL *E. coli*) at the Federal Institute for Risk Assessment (BfR) in Berlin, Germany and from the French Agency for Food, Environmental and Occupational Health and Safety (Anses) in Maisons-Alfort, France. *E. coli* strains used for specificity study included in particular the *E. coli* reference strains belonging to serogroups O1-O181 and H-types H1-H56 (Orskov and Orskov, 1984; Edwards and Ewing, 1986). All strains have been previously described for their serotypes and for virulence genes associated with STEC (Beutin et al., 2015a,b). All strains were grown overnight at 37°C in Luria broth, and DNA was extracted according to manufacturers instructions using InstaGene matrix (BioRad laboratories, Marnes-La-Coquette, France).

Real-time PCR assays were performed with an ABI 7500 instrument (Applied Biosystems, Foster City, CA, USA) in 25- μl reaction volumes, a LightCycler Nano (Roche Diagnostics, Meylan, France) in 10 μl reaction volumes or with a LightCycler 1536 (Roche Diagnostics, Meylan, France) in 1.5-μl reaction volumes according to the recommendations of the suppliers. Primers and TaqMan probes were used at 300 nM final concentrations. The following thermal profile was applied to all instruments: enzyme activation at 95°C for 1–10 min as recommended followed by 40 cycles of denaturation at 95°C and annealing at 60°C.

### PCR detection and mapping of *E. coli* O-antigen and H-antigen genes

Mapping of *fliC* gene variants to their respective H-types was performed as previously described (Beutin et al., 2015a,b). Nucleotide sequence data obtained from thirteen *fliC*_H1_ and eight *fliC*_H12_ genes were used for designing TaqMan® real-time PCR probes and XS probes (minor groove binder replacement, Biolegio, Nijmegen, The Netherlands) and primers for specific detection of all genetic variants of thirteen *fliC*_H1_ and eight *fliC*_H12_ genes (this work). Real-time PCR probes and primers used in this work were designed with the software Primer Express V3.0 (Applied Biosystems) and are described in Table 1.

**Table 1:** **Primers and probes for real-time PCR detection of *E. coli* flagellar types H1 and H12**.

**Target gene**	**Forward primer, reverse primer and probe sequences (5′–3 ′)^a^**	**Length and location within 21 sequences listed in Table 2 (5′ –3′)**
*fliC*_H1_	AGGACGAAATCAAATCCCGTCT	338–359^b^
	ACGGTTCGATGAAAATTCAGGTT	422–444^c^
	[6FAM]- GACCGCGTATCC GGTCA-[BHQ1]^a^	370–386^d^
*fliC*_H12_	TCCATTCAGGACGAAATCAAATC	331–353^b^
	CGTGAACGTACTGGCGAAAG	402–421^e^
	[6FAM] -GTATCTGGCC AGACCCA-[BHQ1]^a^	376–392^f^
*fliC*_H1∕H12_	TGATGGTGAAATGACTACAATTGGT	1329–1353^g^
	GGTAACTGTTGATTCTGGAACTGGT	1395–1419^g^
	[6FAM]–CGAAGTATTCAATCG ATGCTAACAACGGCA–[BHQ1]	1363–1393^g^

a *XS probes (MGB-replacement) were used for fliC_H1_ and fliC_H12_ specific real-time PCRs*.

b *Forward primer conserved in all analyzed fliC_H1_ and fliC_H12_ sequences*.

c *FliC_H1_ reverse primer: one mismatch at position 429: fliC_H1_ = G, fliC_H12_ = A (underlined)*.

d *FliC_H1_ probe: one mismatch at position 381: fliC_H1_ = C, fliC_H12_ = T; and position 384: fliC_H1_ = T, fliC_H12_ = C (underlined)*.

e *FliC_H12_ reverse primer: one mismatch at position 402 with fliC_H1_ = T (4/13 strains), fliC_H12_ = C (underlined)*.

f *FliC_H12_probe: one mismatch at position 381: fliC_H12_ = T, fliC_H1_ = C; and position 384: fliC_H12_ = C, fliC_H1_ = T (underlined)*.

g *Conserved in all 21 fliC_H1_ and fliC_H12_ sequences from Table 2*.

### Nucleotide sequencing

The nucleotide sequence of the PCR products were determined as described (Beutin et al., 2015b) and analyzed with the Accelrys DS Gene software package (Accelrys Inc., USA). The nucleotide sequences of the respective products for *fliC* homologs were determined and have been submitted to European Nucleotide Archive (ENA). The GenBank Accession numbers are listed in Table 2.

**Table 2:** ***Escherichia coli* strains used for nucleotide sequencing of *fliC*_H1_ and *fliC*_H12_ genes and corresponding sequences obtained from GenBank**.

**Strain**	**Serotype**	***fliC* gene GenBank accession no**	**Pathotype**	**Source and References**
CB11962	O20:H12	LN877748^e^	STEC	Calves feces, Germany, 2009, this work
CB13385	O9:K9:H12	LN877749^e^	No data	Chicken meat, Germany 2011, this work
Bi316-42	O9:K9:H12	AY249997	ExPEC	Orskov and Orskov, 1984; Wang et al., 2003
NX9861	O157:H12	AY337474	No data	China, 2003, unpublished
90	O157:H12	AY337471	No data	China, 2003, unpublished
CB11070	O1:H12	LN877750^e^	No data	Pig feces, Germany, 2007, this work
CB12026 (07QMA185.1)	O153:H12	LN877751^e^	STEC	Beef, France, 2009, this work
CB12530	O55:H12	LN877752^e^	STEC	Martin and Beutin, 2011
Su1242	O2:K2:H1	AB028471	ExPEC	Orskov and Orskov, 1984
CFT073	O6:H1	AE014075	ExPEC	Welch et al., 2002
ABU83972	O25:H1	CP001671^a^	ExPEC	Zdziarski et al., 2010
CB13658	O6:H1	LN877753^e^	No data	Pig intestine, Germany, 2011, this work
ATCC25922	O6:H1	CP009072	Human, clinical	Minogue et al., 2014
NRG 857C	O83:H1	CP001855	AIEC	Allen et al., 2008
LF82	O83:H1	CU651637^b^	AIEC	Martinez-Medina et al., 2009
Ec614	O157:H1	JF308285^c^	Beef, no data	Goulter et al., 2010
CB13179	O15:H1	LN877754^e^	Human, ESBL-producer^d^	Geser et al., 2012
CB295	O139:H1	LN877755^e^	STEC	Hampson et al., 1988
CB13050 (D3648)	O139:H1	LN877756^e^	STEC	Scheutz et al., 2012
CB13107	O139:H1	LN877757^e^	STEC	Switzerland, pig intestine, 2011, this work
CB15303	O139:H1	LN877758^e^	STEC	France, edema disease pig, 2014, this work

a *The whole genome sequence of the E. coli strain ABU 83972 (GenBank: CP001671.1) is available (Zdziarski et al., 2010). The O-serogroup of this strain was not reported but its wzx gene (position 2372093–2373353) is >99% similar to wzx of E. coli O25 strains E47a (GenBank GU014554.1) (Wang et al., 2010). Therefore, we classified ABU 83972 here as an O25:H1 strain*.

b *The whole genome sequence of E. coli strain LF82 is available (GenBank: CU651637.1). The O-serogroup of this strain is not reported but its wzx gene (position 2127428–212804) is identical to the wzx gene of E. coli O83:H31 strain H17a GenBank: KJ778808.1 (unpublished) and of E. coli O83:H1 strain NRG857c (GenBank: CP001855.1) (Allen et al., 2008). Therefore, we suggest that LF82 is an O83:H1 strain*.

c *The fliC sequence deposited under GenBank JF308285 is derived from strain EC614 reported as O157:H1 (Goulter et al., 2010). By Blast search, it is 100% identical to the fliC sequence of the H1 reference strain Su1242 (GenBank accession AY249997). Therefore, the flagellar type of EC614 was classified as H1*.

d *Multiresistant, extended-spectrum-lactamase (ESBL)-producing E. coli from healthy human carrier*.

e *The fliC sequence was determined in this study*.

## Results

### Sources and properties of *E. coli* H1 and H12 strains

The *E. coli* H1 and H12 strains investigated in this study were from human, animal, food, and environmental sources (Table 3). The thirty-one flagellar type H1 strains were associated with 10 different *E. coli* O-serogroups, O-rough and O-untypable strains and originated from healthy and diseased humans and animals and from food. The thirty-eight H12 strains divided into thirteen different O-groups of *E. coli*, and in O-untypable and O-rough strains. The H12 strains were from healthy and diseased humans and animals, from food and the environment. Production of Shiga-toxins (Stx) was found in 16 (42.1%) of the H12 strains and associated with five different O-groups. Fourteen (45.2%) of the *E. coli* H1 strains produced Stx, however most of these were from pigs with edema disease (O139:H1, Or:H1) and harbored the *stx2e* gene. O:H types known to be associated with *E. coli* causing extraintestinal infections of humans (O2:H1, O4:H1, O6:H1, O25:H1) were detected among the investigated H1 strains. Interestingly, strains belonging to these serotypes originated not only from humans but also from animals and food. Certain strains belonged to serotypes which have not been previously associated with clinical disease and their role of pathogens for humans and animals is not yet known.

**Table 3:** **Source and origin of *E. coli* H1 and H12 strains**.

**Serotype^a^**	**Nos. of strains**	**Source**	**Origin/References**
O2:H1	1	Calves feces, diarrhea	Germany, 2010
O2:K2:H1	1	Human blood	Orskov and Orskov, 1984
O4:H1	1	Raw milk cheese	Germany, 2010
O6:H1	4	Pig feces, diarrhea (1), Human (3)	Germany, 2011 Germany, 2009
O6:K5:H1	1	Human feces	Reister et al., 2014
O15:H1	3	Rabbit (2) Human (1)	Switzerland, 2007 Geser et al., 2012
O22:H1	2	Goat cheese (1) Human peritoneum (1)	Germany, 2013 Orskov and Orskov, 1984
O25:H1	1	Dog feces	Germany, 2011
O77:H1	1	salad	Germany, 2009
O79:H1	1	hare	Germany, 2007
O139:H1	11^b^	Pig feces/organs, edema disease Wild boar feces/organs, edema disease	Orskov and Orskov, 1984: Beutin et al., 2008 France, 2013
O149:H1	2^c^	Beef	Germany, 2011
ONT:H1	1	human	Germany, 2011
Or:H1	1^b^	Pig feces, edema disease	Germany, 2014
O1:H12	1	Pig feces	Germany 2007
O9:K9:H12	5	Human peritoneum (1) Surface water (3) Chicken meat (1)	Orskov and Orskov, 1984 Germany, 2013 Germany, 2013
O9:H12	4	Pork	Martin and Beutin, 2011
O11:H12	2	Human (1) Pig feces (1)	Geser et al., 2012 Germany, 2009
O20:H12	2^d^	Calves feces/organs, diarrhea	Germany, 2009
O49:H12	1	Human urine	Orskov and Orskov, 1984
O55:H12	2^e^	Milk, beef	Martin and Beutin, 2011
O79:H12	1	Surface water	Germany, 2011
O98:H12	1	Pork	Germany, 2013
O104:H12	2	Human, diarrhea Surface water	Miko et al., 2013 Germany, 2013
O118:H12	3^d^	Human, diarrhea	Pierard et al., 1998; Beutin et al., 2004
O136:H12	4^e^	Milk Cattle feces	Martin and Beutin, 2011 France, 1998 Canada, 2012
O153:H12	3^e^	beef	Martin and Beutin, 2011
O157:H12	3	Human Pig	Germany, 2007 Kaufmann et al., 2006
ONT:H12	2	Milk Surface water	Germany, 2014 Germany, 2013
Or:H12	2^e^	Cattle feces	Germany, 2010

a *This list includes serotype reference strains: Nissle 1917 (O6:K5:H1) (Reister et al., 2014), EH250 (O118:H12), (Scheutz et al., 2012), Su 1242 (O2:K2:H1), E14a (O22:H1), CDC 63-57 (O139:H1), Bi316-42 (O9:K9:H12), U12-41 (O49:H1) (Orskov and Orskov, 1984)*.

b *Positive for stx2e*.

c *Positive for stx1d*.

d *Positive for stx2*.

e *Positive for stx1*.

### Nucleotide analysis of *E. coli fliC*_H1_ and *fliC*_H12_ genes

The nucleotide sequences of the reference strains (Orskov and Orskov, 1984) for *E. coli* flagellar antigens H1 (strain Su1242, GenBank accession AB028471.1) and H12 (Bi 316-42, GenBank accession AY249997) (Wang et al., 2003) have been published previously. The length of coding sequence of each *fliC*_H1_ and *fliC*_H12_ gene is 1788 nucleotides and both sequences have 97.5% identity (44 nucleotide exchanges) at the nucleotide level and 98.98% identity and 99.16% similarity at the amino acid level (7 amino acids (aa) exchanges). Additional *fliC* nucleotide sequences from six *E. coli* H1 and five *E. coli* H12 strains were obtained in this work (Table 2). These sequences were compared with seven *fliC*_H1_ sequences and three *fliC*_H12_ sequences already available in GenBank (Table 2). All 21 H1 or H12 flagellin genes have a 1788 nucleotides length that codes for 595 amino acid residues.

**Figure 1:**
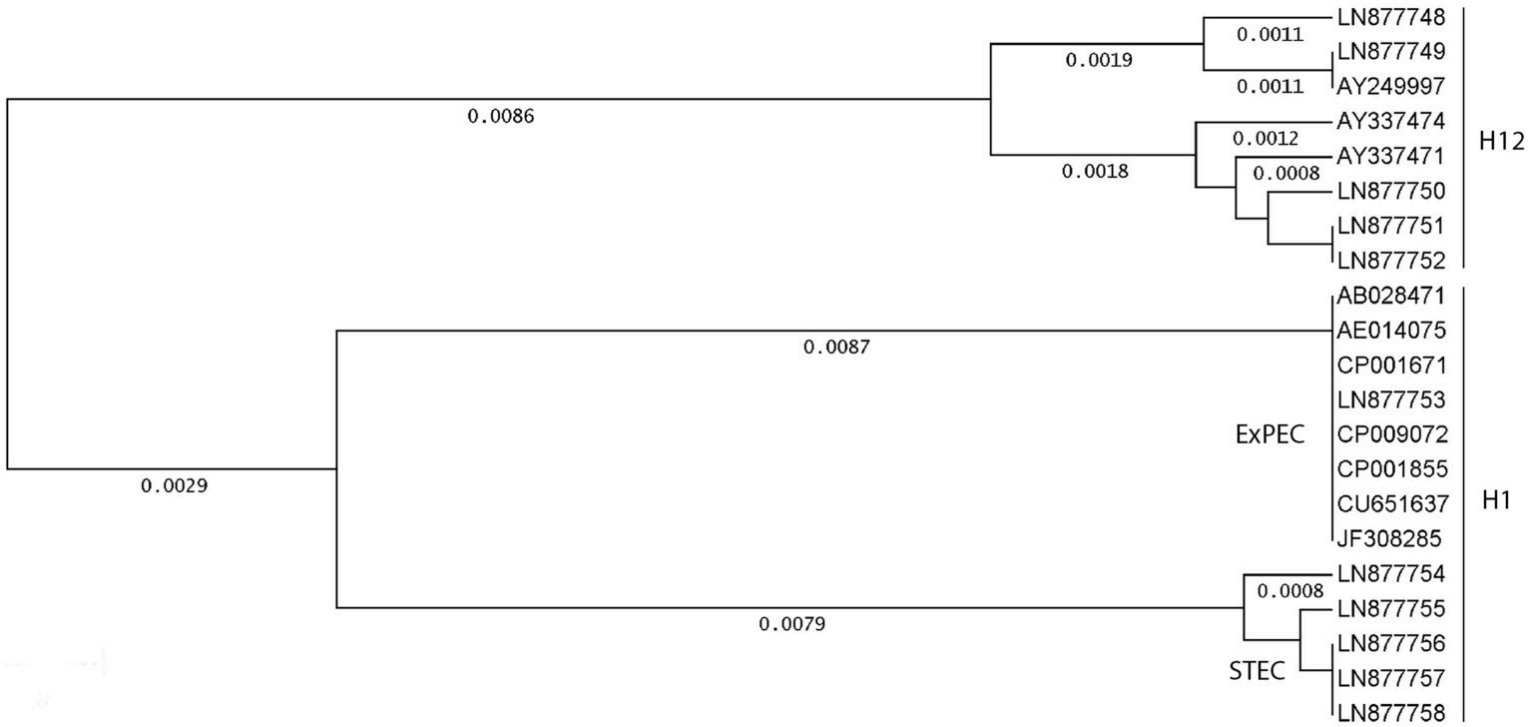
**Genetic relationships between *fliC*_H1_ and *fliC*_H12_ genes in different strains and serotypes of *E. coli***. Cluster analysis was performed using eight *fliC*_H12_ and thirteen *fliC*_H1_ genes listed in Table 2. GenBank accession numbers are indicated for orientation. The unweighted-pair group method using average linkages was used as a tree-building mode, and the distances were calculated according to Tajima and Nei (1984) using the Accelrys DS Gene software package.

A cluster analysis performed with thirteen *fliC*_H1_ and eight *fliC*_H12_ sequences is shown in Figure 1. Four different genotypes were detected among the thirteen *fliC*_H1_ strains. Uropathogenic *E. coli* O2:H1, O6:H1, O25:H1, and AIEC O83:H1 strains were identical for their *fliC*_H1_ sequences and assigned to a large cluster composed by eight strains. A smaller cluster was formed by five *fliC*_H1_ strains; four of these were Stx2e producing O139:H1 causing edema disease in pigs.

Six different genotypes were found among the eight *fliC*_H12_ strains. Identical *fliC*_H12_ sequences were only found between two O9:K9:H12 strains and each one O55:H12 and O153:H12 strain, respectively.

### Amino acid alterations between flagellar antigens H1 and H12 in *E. coli* strains

**Figure 2:**
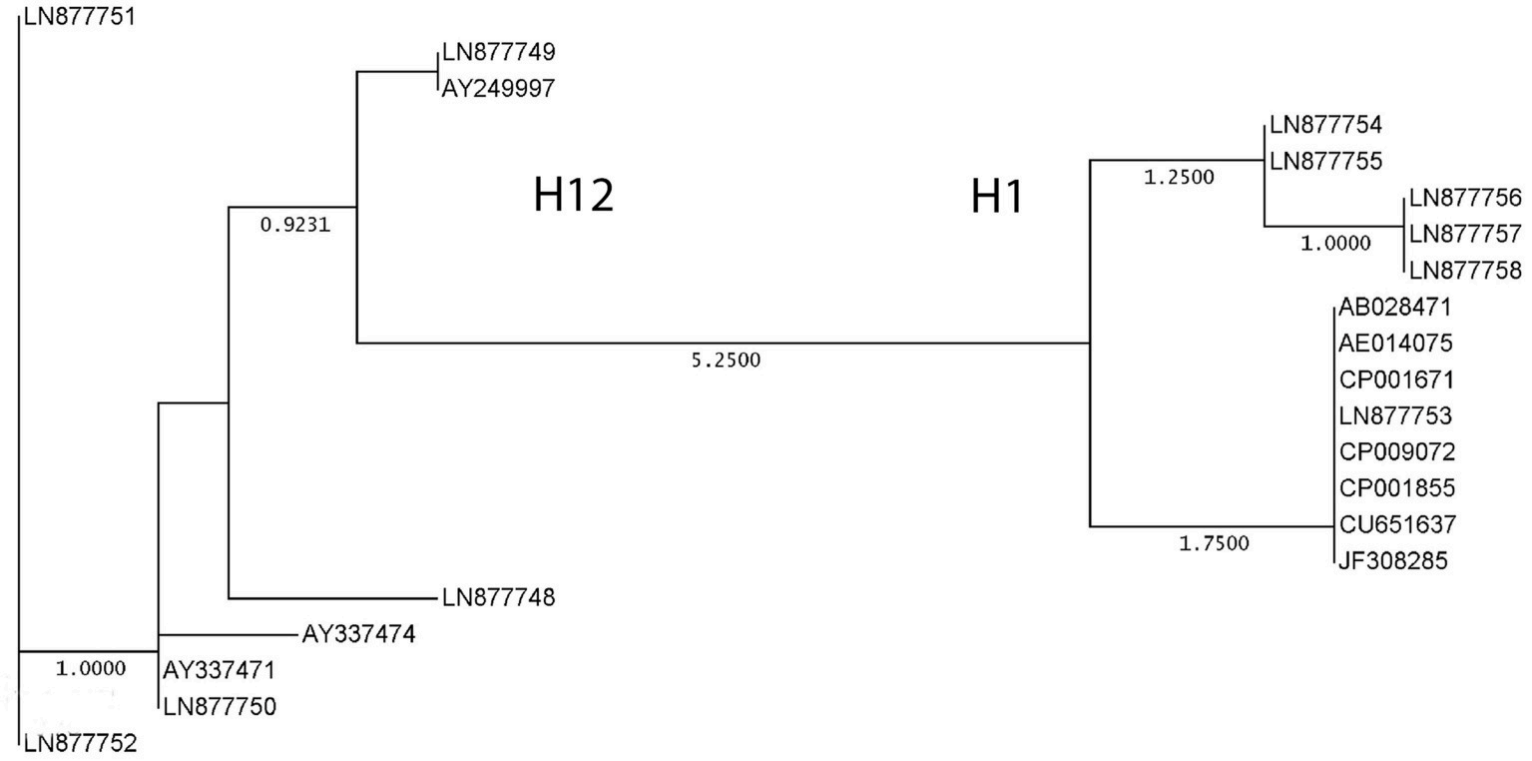
**Genetic relationships between translation products of thirteen *fliC*_H1_ and eight *fliC*_H12_ genes in different strains and serotypes of *E. coli* listed in Table 2**. GenBank accession numbers are indicated for orientation. The neighbor joining method with absolute differences (best tree) was used as a tree-building mode (Nei, 1996) using the Accelrys DS Gene software package.

An alignment of the amino acid sequences of thirteen *fliC*_H1_ and eight *fliC*_H12_ strains is shown in Table S1. All translation products had a length of 595 amino acids (aa). The eight *fliC*_H12_ strains were showing only few alterations with one or more of the strains at aa positions 249, 258, 339, and 472 (99.2% similarity) (Table S1), generating six different protein sequences (Figure 2). The thirteen *fliC*_H1_ strains split into three protein sequences (Figure 2) differing at positions 258, 431, and 481 (99.5% similarity) (Table S1). The aa changes were all located in the variable region of *fliC* encoding flagellar antigen specificity (Wang et al., 2003). Differences in the aa sequence which could distinguish between all investigated *fliC*_H1_ and *fliC*_H12_ strains, respectively, were found at positions 302 (Glu/Lys), 340 (Asn/Lys), 361 (Gly/Asp), 391 (Thr/Lys), 396 (Asn/Asp), and 430 (Asn/Lys). The six flagellar type specific aa sequence differences were all located in the variable region of the *fliC*_H1_ and *fliC*_H12_ genes.

### Development and evaluation of real-time PCR assays for identification of *E. coli fliC*_H1_ and *fliC*_H12_ strains

The close similarity between *E. coli fliC*_H1_ and *fliC*_H12_ translation products explains the serological cross-reactivity which was previously described for H1 and H12 antigens (Orskov and Orskov, 1984; Edwards and Ewing, 1986). As specific differences were found that distinguish between *fliC*_H1_ and *fliC*_H12_ sequences, molecular detection of the respective *fliC* genes could be more suitable than serotyping for clear identification of H1 and H12 strains of *E. coli*.

Based on the sequence data obtained for *E. coli fliC*_H1_ and *fliC*_H12_ genes we developed a TaqMan real-time PCR assay for common detection of *fliC*_H1_ and *fliC*_H12_ genes as well as real-time PCR assays for specific detection of *fliC*_H1_ and *fliC*_H12_, respectively (Table 1). Short-length XS-probes (minor groove binder replacement) had to be employed to develop real-time PCR assays specific for *fliC*_H1_ and *fliC*_H12_ sequences (Table 1). We used two nucleotide substitutions between the sequences of *fliC*_H1_ and *fliC*_H12_ to design specific probes (Table 1).

The assays were first tested for sensitivity and specificity on 31 *E. coli* H1 and 38 *E. coli* H12 strains (Table 4) as well as on the *E. coli* H-type reference strains (H1-H56) (Orskov and Orskov, 1984; Edwards and Ewing, 1986). The real-time PCR for common detection of *fliC*_H1_ and *fliC*_H12_ reacted with all tested *E. coli* H1 and H12 strains (Table 4) and not with any of the reference strains encoding all other flagellar antigens than H1 and H12.

**Table 4:** **Detection of different *E. coli* H1 and H12 strains belonging to different O-serogroups by *fliC*_H1_, *fliC*_H12_ and *fliC*_H1/H12_ Real Time PCR assays**.

**Serotype^b^**	**Nos. of strains**	**CT-values^a^*fliC*_H1_**	**CT-values^a^*fliC*_H12_**	**CT-values^a^*fliC*_H1/H12_**
O2:H1	1	21.1	–	22.2
O2:K2:H1^b^	1	21.1–24.3	–	22.2–22.9
O4:H1	1	24.7	–	27.1
O6:H1	5	21.1–24.3	–	21.9–25.3
O15:H1	3	19.6–25.5	–	21.2–24.0
O22:H1	2	19.6–22.0	–	22.6–24.2
O25:H1	1	22.5	–	24.5
O77:H1	1	21.8	–	23.1
O79:H1	1	24.5	–	24.7
O139:H1	11	16.5–23.7	–	15.0–24.6
O149:H1	2	213–21.4	–	23.5–24.8
ONT:H1	1	22.6	–	22.7
Or:H1	1	20.1	–	21.9
O1:H12	1	–	20.9	24.2
O9:K9:H12^b^	5	–	18.6–23.2	21.6–23.1
O9:H12	4	–	17.0–17.9	16.0–22.6
O11:H12	2	–	18.2–22.4	21.7–22.6
O20:H12	2	–	15.2–16.9	20.4–21.1
O49:H12	1	–	19.6	24.5
O55:H12	2	–	20.1–22.8	22.5–22.7
O79:H12	1	–	22.8	23.0
O98:H12	1	–	22.6	24.3
O104:H12	2	–	20.9–23.6	22.1–23.4
O118:H12	3	–	18.0–23.1	17.1–23.4
O136:H12	4	–	16.8–23.6	15.9–23.2
O153:H12	3	–	18.3–20.9	22.2–23.2
O157:H12	3	–	18.7–23.4	21.7–23.7
ONT:H12	2	–	21.7–22.8	22.5–23.4
Or:H12	2	–	19.2–20.7	23.0–23.3

a *Range of real time PCR cycle thresholds. Negative reactions are indicated with the “–” sign*.

b *Reference strain Orskov non-motile and the fliC-genotype was detected by nucleotide sequencing of fliC PCR products*.

The *fliC*_H1_ and *fliC*_H12_ gene specific assays detected all *E. coli* H1 and all *E. coli* H12 strains, respectively (Table 4). However, both assays showed cross-reactions with some flagellar type reference strains different from H1 and H12. With the *fliC*_H1_ real-time PCR, cross-reactions were observed with H6, H7, H15, H20, H34, H37, H41, H45, H46, H49, and H52 strains. The *fliC*_H12_ specific PCR reacted also with H7, H28, H31, and H41 strains (Table 5). Although the overall sequences of the *fliC* genes of H-types cross-reacting with the *fliC*_*H*1_ and *fliC*_*H*12_ real-time PCR are widely different from those of *fliC*_*H*1_ and *fliC*_*H*12_, they show high local similarities with the primers and probes sequences. In cases of cross reactivity, no or only minor differences (0–3 mismatches) were found between target-sequences and *fliC*_H1_ and *fliC*_H12_, primers and probes (Table 5). Three and more mismatches were found in cases of negative real-time PCR results.

**Table 5:** **Cross-reactions of *fliC*_H1_ and *fliC*_H12_ real time PCR assays with other flagellar types of *E. coli***.

**Reference strain^a^**	**H-type**	**GenBank Accession No**.	**Detector tested^b^**	**Ct-value^c^**	**Mismatch FP/P/RP^d^**
A20	H6	AY249991.1	*fliC*_H1_	26.8	0/0/0
			*fliC*_H12_	–	0/2/1
U5-41	H7	AB028474.1	*fliC*_H1_	26.0	0/1/0
			*fliC*_H12_	24.5	0/1/1
E39a	H15	AY249999.1	*fliC*_H1_	28.2	1/1/1
			*fliC*_H12_	–	3/2/2
H3306	H20	AY250003.1	*fliC*_H1_	27.0	0/0/1
			*fliC*_H12_	–	2/2/2
HW30	H28	AY337469.1	*fliC*_H1_	–	0/2/1
			*fliC*_H12_	21.9	0/0/0
HW33	H31	AF345849.1	*fliC*_H1_	–	0/2/4
			*fliC*_H12_	22.9	0/0/0
BP 12665	H34	AY250016.1	*fliC*_H1_	20.9	0/0/0
			*fliC*_H12_	–	0/2/1
P11a	H37	AY250017.1	*fliC*_H1_	26.4	1/0/1
			*fliC*_H12_	–	3/2/1
RVC1787	H41	AY250020.1	*fliC*_H1_	27.1	0/1/1
			*fliC*_H12_	24.0	0/1/1
4106-54	H45	AY250023.1	*fliC*_H1_	25.5	0/0/0
			*fliC*_H12_	–	0/3/1
5306-56	H46	AY250024.1	*fliC*_H1_	27.6	0/0/1
			*fliC*_H12_	–	0/1/2
2147-59	H49	AY250026.1	*fliC*_H1_	24.6	0/0/0
			*fliC*_H12_	–	0/3/1
C2187-69	H52	AY250028.1	*fliC*_H1_	26.8	0/1/1
			*fliC*_H12_	–	0/3/1
Su1242	H1	AB028471.1	*fliC*_H1_	21.1–24.3	0/0/0
			*fliC*_H12_	–	0/2/1
Bi316/42	H12	AY249997.1	*fliC*_H1_	–	0/2/1
			*fliC*_H12_	18.6–23.6	0/0/0

a *H-type reference strains (Orskov and Orskov, 1984)*.

b *As listed in Table 1*.

c *Mean of real-time cycle threshold (CT-values) calculated from duplicate PCRs. Negative reactions are indicated with the “–” sign*.

d *Number of mismatches found between real-time detector sequence and target gene sequence. FP, forward primer; P, gene probe; RP, reverse primer*.

In respect to these findings, the assays were then tested on a second panel of 78 strains comprising strains with H-types previously found to cross-react with FlicH1 or FlicH12 PCR assays as well as strains from O-groups that can be found associated with H1 and H12, but with H-types different from H1 and H12 (Table 6).

**Table 6:** **Reaction of the *fliC*_H1/H12_, *fliC*_H1_ and *fliC*_H12_ real-time PCR assays with non-H1 and non-H12 strains**.

**Serotype**	**Number of strains**	**Ct-values^a^*fliC*_H1/H12_**	**Ct-values^a^*fliC*_H1_**	**Ct-value^a^*fliC*_H12_**
O33:H6	1	–	20.6	–
O40:H6	1	–	19.35	–
O55:H6	1	–	17.2	–
O63:H6	1	–	18.98	–
O113:H6	2	–	19.38–19.46	–
O125:H6	1	–	24.29	–
O126:H6	1	–	20.17	–
O127:H6	1	–	21.78	–
O41:H7	1	–	–	21.11
O55:H7	3	–	–	22.35–26.16
O153:H7	1	–	–	18.21
O157:H7	4	–	–	18.46–24.7
O23:H15	1	–	–	–
O157:H15	1	–	–	–
O28:H28	1	–	–	18.73
O91:H28	1	–	–	16.34
O110:H28	1	–	–	15.53
O116:H28	1	–	–	30.55
O145:H28	1	–	–	14.99
OX185:H28	1	–	–	17.18
O51:H49	1	–	21.1	–
O114:H49	1	–	20.65	–
O181:H49	1	–	20.6	–
O45:H31	1	–	19.44	–
O179:H31	1	–	–	19.56
O6:H34	2	–	–	–
O86:H34	1	–	21.4	–
O142:H34	1	–	21.99	–
O145:H34	1	–	–	21.67
O132:H34	1	–	–	–
O132:H34	1	–	–	20.84
O76:H41	1	–	–	20.75
O17/77:H41	1	–	21.93	–
O8:H45	1	–	–	–
O121:H45	1	–	22.32	–
O157:H45	1	–	24.34	–
O186:[H45]	1	–	20.92	–
O119:[H52]	1	–	–	–
O2:H8	1	–	–	–
O2:H25	1	–	27.15	–
O2:H27	1	–	–	–
O2:H40	1	–	–	–
O4:H5	1	–	–	–
O4:H16	1	–	–	–
O6	2	–	–	–
O6:H4	1	–	24.17	–
O6:H10	1	–	–	–
O7:H4	1	–	–	–
O15:H2	1	–	–	–
O15:H11	1	–	–	–
O15:H16	1	–	–	–
O15:H21	1	–	–	–
O139:H4	1	–	–	–
O139:H19	1	–	–	–
O128:H2	1	–	–	–
O128:H8	1	–	–	–
O20:H9	1	–	–	23.23
O20:H30	1	–	–	–
O20:H33	1	–	–	–
O20:NM	1	–	–	–
O55:H19	1	–	–	23.43
O55:H21	1	–	–	–
O55:H51	1	–	–	–
O118:H2	1	–	–	–
O118:H5	1	–	–	–
O118:H8	1	–	–	–
O118:H16	1	–	–	–
O153:H14	1	–	–	20.86
O153:H21	1	–	–	–
O153:H25	1	–	28.37	–

a *Range of real time PCR cycle thresholds. Negative reactions are indicated with the “–” sign*.

None of the 78 strains with H–types different from H1 and H12 reacted with the common *fliC*_H1_ / *fliC*_H12_ real-time PCR assay. Cross reactions with the *fliC*_H1_ real-time PCR-assay were observed with H6 (9/9), H49 (3/3), H31 (1/2), H34 (2/7), H41 (1/2), H45 (3/4) as well as with one O6:H4 strain. Weak cross-reactions were also observed with one O2:H25 strain and one O153:H25 strain. Cross-reactions with the *fliC*_H12_ real-time PCR-assay were observed with H7 (9/9), H28 (6/6), H31 (1/2), H34 (2/7), H41 (1/2), as well as one O20:H9, one O55:H19 and one O153:H14 strains. In contrast to the respective reference strains, cross-reactions were not observed with either real-time PCR-assay with two other H15 and one H52 strain tested (Tables 5, 6). We do not know if these three strains show further differences in the PCR-target region which could explain these findings.

Overall, molecular typing of *E. coli* H1 and H12 strains requires first identification of H1/H12 strains with the common *fliC*_H1_/*fliC*_H12_ real-time PCR assay, followed by specific identification of *fliC*_H1_ and *fliC*_H12_, by their respective real-time PCR-assays. The real-time PCR for common detection of *fliC*_H1_ and *fliC*_H12_ was found 100% sensitive and 100% specific. The *fliC*_H1_ and *fliC*_H12_ gene specific assays were found 100% sensitive as they detected all *E. coli* H1 and all *E. coli* H12 strains, respectively. When used exclusively on H1 and H12 strains (as identified by the common primers/probe set in a first step), the *fliC*_H1_ and *fliC*_H12_ gene specific assays were found 100% specific. Thus, 100% of H1 and H12 strains would be accurately typed with this system.

## Discussion

The genetically and serologically closely related flagellar antigens H1 and H12 were found in heterogeneous types of *E. coli* strains belonging to 26 different O-serogroups, O-untypable and O-rough strains. With one exception (O79:H1 and O79:H12), H1 and H12 strains did not share common O-serogroups which would indicate that flagellar types H1 and H12 have separated from each other not very recently in evolution. They may have evolved independently following rearrangements in the O-group loci of ancestor strains carrying the closely related H1/H12 flagellar types and do not directly derive from a common O-group ancestor.

By comparing nucleotide sequences of *fliC* genes from thirteen H1 and eight H12 strains we identified six H-type specific aa changes at positions 302, 340, 361, 391, 396, and 430. All these are located in the variable part of flagellin determining antigen specificity (Wang et al., 2003). As these changes are characteristic for the respective flagellar antigen, we suppose them to determine the antigen specificities of H1 and H12. The few other aa changes detected in some H1 and H12 strains might thus not be significant as specific characteristics of H1 or H12 types. However, such aa-changes could explain the finding of serological subtypes of H1 which were detected using factor specific H-antisera (Ratiner et al., 1995).

Interestingly, the genetic distance between *fliC*_H1_ (Su1242, GenBank accession AB028471.1) and *fliC*_H12_ sequences (Bi 316-42, GenBank accession AY249997) (97.5% similarity) is less than that found between different alleles of *fliC*_H28_ (92.0% similarity) (Beutin et al., 2015b). It is slightly bigger than the distance found among different alleles of *fliC*_H19_ (98.5% similarity) (Beutin et al., 2015a). Multiple allelelic types of *fliC* were also detected in *E. coli* H6, H7, H8, H25, and H40 strains, respectively (Reid et al., 1999; Wang et al., 2000; Beutin and Strauch, 2007; Beutin et al., 2015b). Already, a considerable number of serological cross-reactions were observed when flagellar types H1–H56 were compared (Orskov and Orskov, 1984; Edwards and Ewing, 1986). Some of these flagellar antigens (H1/H12, H8/H40, H11/H21, and H37/H41) are so closely related that the use of cross-absorbed antisera is needed to obtain unambiguous serotyping results (Edwards and Ewing, 1986).

The presence of allelic subtypes within *fliC* genes encoding different H-types of *E. coli* and the finding that different flagellar types are serologically cross-reacting may complicate *E. coli* strain typing using H-antisera. The use of molecular typing procedures, such as real-time PCR can solve the typing problem caused by serologically closely related H-antigens, as we have shown for H1 and H12 in this work. Using primer express V3.0 software, it was not possible to design real-time PCRs specific exclusively for *fliC*_H1_ and *fliC*_H12_, respectively. For this reason, we employed a two-step real-time detection procedure. The first step uses a real-time PCR highly specific for both H1 and H12 strains, followed by subtyping of H1/H12-positive strains with the respective *fliC*_H1_ and *fliC*_H12_ specific real-time PCRs. Short probe sequence lengths as obtained with minor groove binder (MGB) or MGB-replacements (XS-probe) are needed to ensure specificity between closely similar DNA-targets as previously shown for *fliC*_H19_ allelic discrimination (Beutin et al., 2015a). The PCRs could be used in parallel for examination of large number of isolates using high throughput PCR platforms as described previously for analysis of large numbers of *Clostridia* and *E. coli* strains (Delannoy et al., 2013; Woudstra et al., 2013).

Unambiguous typing of *fliC*_H1_ and *fliC*_H12_ sequences is of interest for clinical and epidemiological investigations since some H1 and H12 strains were shown to play a role as pathogens in humans and animals.

More than one third of investigated H1 and H12 strains produced Shiga toxins. Strains showing O:H types characteristic for ExPEC associated with human diseases (O2:H1, O4:H1, O6:H1, O15:H1) were detected in this work. Interestingly, these were not only from humans but also found in animals and food. It was previously described that animals, food and water can be a source of pandemic ExPEC strains (Jakobsen et al., 2010; Riley, 2014; Gomi et al., 2015; Singer, 2015). Flagellar type H12 strains encompass mainly STEC (O20:H12, O55:H12, O118:H12, O136:H12, O153:H12, and Or:H12) and were isolated from diseased animals and humans, food and the environment (Scheutz and Strockbine, 2005).

The specific molecular detection of H1 and H12 flagellins as described in this study will be useful for diagnosis and for source attribution of human and animal pathogenic ExPEC and STEC strains in outbreaks and sporadic cases of infection.

## Author contributions

Conceived and designed the experiments: LB, SD, PF. Performed the experiments: LB, SD. Analyzed the data: LB, SD, PF. Contributed reagents/materials/analysis tools: LB, SD, PF. Wrote the paper: LB, SD, PF. Critical revision of the paper for important intellectual content: LB, SD, PF.

## Funding

The project was partially financed by the French “joint ministerial program of R&D against CBRNE risks” (Grant number C17609- 2).

### Conflict of interest statement

The authors declare that the research was conducted in the absence of any commercial or financial relationships that could be construed as a potential conflict of interest.
