# Supplementary material for: Genetic Analysis and Detection of fliCH1 and fliCH12 Genes Coding for Serologically Closely Related Flagellar Antigens in Human and Animal Pathogenic Escherichia coli
Source: Front Microbiol. 2016 Feb 15;7:135. doi: 10.3389/fmicb.2016.00135 (PMC4753304; doi:10.3389/fmicb.2016.00135)
Supplement: Supplementary file 1 [file Table1.PDF]

Alignment Name: Table S1 AA alignmentUntitled5.aaa_bsml
Length: 595

										10										20										30										40						
LN877748	M	A	Q	V	I	N	T	N	S	L	S	L	I	T	Q	N	N	I	N	K	N	Q	S	A	L	S	S	S	I	E	R	L	S	S	G	L	R	I	N	S	A	K	D	D	A	A
LN877749	M	A	Q	V	I	N	T	N	S	L	S	L	I	T	Q	N	N	I	N	K	N	Q	S	A	L	S	S	S	I	E	R	L	S	S	G	L	R	I	N	S	A	K	D	D	A	A
AY249997	M	A	Q	V	I	N	T	N	S	L	S	L	I	T	Q	N	N	I	N	K	N	Q	S	A	L	S	S	S	I	E	R	L	S	S	G	L	R	I	N	S	A	K	D	D	A	A
AY337474	M	A	Q	V	I	N	T	N	S	L	S	L	I	T	Q	N	N	I	N	K	N	Q	S	A	L	S	S	S	I	E	R	L	S	S	G	L	R	I	N	S	A	K	D	D	A	A
AY337471	M	A	Q	V	I	N	T	N	S	L	S	L	I	T	Q	N	N	I	N	K	N	Q	S	A	L	S	S	S	I	E	R	L	S	S	G	L	R	I	N	S	A	K	D	D	A	A
LN877750	M	A	Q	V	I	N	T	N	S	L	S	L	I	T	Q	N	N	I	N	K	N	Q	S	A	L	S	S	S	I	E	R	L	S	S	G	L	R	I	N	S	A	K	D	D	A	A
LN877751	M	A	Q	V	I	N	T	N	S	L	S	L	I	T	Q	N	N	I	N	K	N	Q	S	A	L	S	S	S	I	E	R	L	S	S	G	L	R	I	N	S	A	K	D	D	A	A
LN877752	M	A	Q	V	I	N	T	N	S	L	S	L	I	T	Q	N	N	I	N	K	N	Q	S	A	L	S	S	S	I	E	R	L	S	S	G	L	R	I	N	S	A	K	D	D	A	A
AB028471	M	A	Q	V	I	N	T	N	S	L	S	L	I	T	Q	N	N	I	N	K	N	Q	S	A	L	S	S	S	I	E	R	L	S	S	G	L	R	I	N	S	A	K	D	D	A	A
AE014075	M	A	Q	V	I	N	T	N	S	L	S	L	I	T	Q	N	N	I	N	K	N	Q	S	A	L	S	S	S	I	E	R	L	S	S	G	L	R	I	N	S	A	K	D	D	A	A
CP001671	M	A	Q	V	I	N	T	N	S	L	S	L	I	T	Q	N	N	I	N	K	N	Q	S	A	L	S	S	S	I	E	R	L	S	S	G	L	R	I	N	S	A	K	D	D	A	A
LN877753	M	A	Q	V	I	N	T	N	S	L	S	L	I	T	Q	N	N	I	N	K	N	Q	S	A	L	S	S	S	I	E	R	L	S	S	G	L	R	I	N	S	A	K	D	D	A	A
CP009072	M	A	Q	V	I	N	T	N	S	L	S	L	I	T	Q	N	N	I	N	K	N	Q	S	A	L	S	S	S	I	E	R	L	S	S	G	L	R	I	N	S	A	K	D	D	A	A
CP001855	M	A	Q	V	I	N	T	N	S	L	S	L	I	T	Q	N	N	I	N	K	N	Q	S	A	L	S	S	S	I	E	R	L	S	S	G	L	R	I	N	S	A	K	D	D	A	A
CU651637	M	A	Q	V	I	N	T	N	S	L	S	L	I	T	Q	N	N	I	N	K	N	Q	S	A	L	S	S	S	I	E	R	L	S	S	G	L	R	I	N	S	A	K	D	D	A	A
JF308285	M	A	Q	V	I	N	T	N	S	L	S	L	I	T	Q	N	N	I	N	K	N	Q	S	A	L	S	S	S	I	E	R	L	S	S	G	L	R	I	N	S	A	K	D	D	A	A
LN877754	M	A	Q	V	I	N	T	N	S	L	S	L	I	T	Q	N	N	I	N	K	N	Q	S	A	L	S	S	S	I	E	R	L	S	S	G	L	R	I	N	S	A	K	D	D	A	A
LN877755	M	A	Q	V	I	N	T	N	S	L	S	L	I	T	Q	N	N	I	N	K	N	Q	S	A	L	S	S	S	I	E	R	L	S	S	G	L	R	I	N	S	A	K	D	D	A	A
LN877756	M	A	Q	V	I	N	T	N	S	L	S	L	I	T	Q	N	N	I	N	K	N	Q	S	A	L	S	S	S	I	E	R	L	S	S	G	L	R	I	N	S	A	K	D	D	A	A
LN877757	M	A	Q	V	I	N	T	N	S	L	S	L	I	T	Q	N	N	I	N	K	N	Q	S	A	L	S	S	S	I	E	R	L	S	S	G	L	R	I	N	S	A	K	D	D	A	A
LN877758	M	A	Q	V	I	N	T	N	S	L	S	L	I	T	Q	N	N	I	N	K	N	Q	S	A	L	S	S	S	I	E	R	L	S	S	G	L	R	I	N	S	A	K	D	D	A	A
	M	A	Q	V	I	N	T	N	S	L	S	L	I	T	Q	N	N	I	N	K	N	Q	S	A	L	S	S	S	I	E	R	L	S	S	G	L	R	I	N	S	A	K	D	D	A	A

Alignment Name: Table S1 AA alignmentUntitled5.aaa_bsml
Length: 595

				50										60										70										80										90		
LN877748	G	Q	A	I	A	N	R	F	T	S	N	I	K	G	L	T	Q	A	A	R	N	A	N	D	G	I	S	V	A	Q	T	T	E	G	A	L	S	E	I	N	N	N	L	Q	R	I
LN877749	G	Q	A	I	A	N	R	F	T	S	N	I	K	G	L	T	Q	A	A	R	N	A	N	D	G	I	S	V	A	Q	T	T	E	G	A	L	S	E	I	N	N	N	L	Q	R	I
AY249997	G	Q	A	I	A	N	R	F	T	S	N	I	K	G	L	T	Q	A	A	R	N	A	N	D	G	I	S	V	A	Q	T	T	E	G	A	L	S	E	I	N	N	N	L	Q	R	I
AY337474	G	Q	A	I	A	N	R	F	T	S	N	I	K	G	L	T	Q	A	A	R	N	A	N	D	G	I	S	V	A	Q	T	T	E	G	A	L	S	E	I	N	N	N	L	Q	R	I
AY337471	G	Q	A	I	A	N	R	F	T	S	N	I	K	G	L	T	Q	A	A	R	N	A	N	D	G	I	S	V	A	Q	T	T	E	G	A	L	S	E	I	N	N	N	L	Q	R	I
LN877750	G	Q	A	I	A	N	R	F	T	S	N	I	K	G	L	T	Q	A	A	R	N	A	N	D	G	I	S	V	A	Q	T	T	E	G	A	L	S	E	I	N	N	N	L	Q	R	I
LN877751	G	Q	A	I	A	N	R	F	T	S	N	I	K	G	L	T	Q	A	A	R	N	A	N	D	G	I	S	V	A	Q	T	T	E	G	A	L	S	E	I	N	N	N	L	Q	R	I
LN877752	G	Q	A	I	A	N	R	F	T	S	N	I	K	G	L	T	Q	A	A	R	N	A	N	D	G	I	S	V	A	Q	T	T	E	G	A	L	S	E	I	N	N	N	L	Q	R	I
AB028471	G	Q	A	I	A	N	R	F	T	S	N	I	K	G	L	T	Q	A	A	R	N	A	N	D	G	I	S	V	A	Q	T	T	E	G	A	L	S	E	I	N	N	N	L	Q	R	I
AE014075	G	Q	A	I	A	N	R	F	T	S	N	I	K	G	L	T	Q	A	A	R	N	A	N	D	G	I	S	V	A	Q	T	T	E	G	A	L	S	E	I	N	N	N	L	Q	R	I
CP001671	G	Q	A	I	A	N	R	F	T	S	N	I	K	G	L	T	Q	A	A	R	N	A	N	D	G	I	S	V	A	Q	T	T	E	G	A	L	S	E	I	N	N	N	L	Q	R	I
LN877753	G	Q	A	I	A	N	R	F	T	S	N	I	K	G	L	T	Q	A	A	R	N	A	N	D	G	I	S	V	A	Q	T	T	E	G	A	L	S	E	I	N	N	N	L	Q	R	I
CP009072	G	Q	A	I	A	N	R	F	T	S	N	I	K	G	L	T	Q	A	A	R	N	A	N	D	G	I	S	V	A	Q	T	T	E	G	A	L	S	E	I	N	N	N	L	Q	R	I
CP001855	G	Q	A	I	A	N	R	F	T	S	N	I	K	G	L	T	Q	A	A	R	N	A	N	D	G	I	S	V	A	Q	T	T	E	G	A	L	S	E	I	N	N	N	L	Q	R	I
CU651637	G	Q	A	I	A	N	R	F	T	S	N	I	K	G	L	T	Q	A	A	R	N	A	N	D	G	I	S	V	A	Q	T	T	E	G	A	L	S	E	I	N	N	N	L	Q	R	I
JF308285	G	Q	A	I	A	N	R	F	T	S	N	I	K	G	L	T	Q	A	A	R	N	A	N	D	G	I	S	V	A	Q	T	T	E	G	A	L	S	E	I	N	N	N	L	Q	R	I
LN877754	G	Q	A	I	A	N	R	F	T	S	N	I	K	G	L	T	Q	A	A	R	N	A	N	D	G	I	S	V	A	Q	T	T	E	G	A	L	S	E	I	N	N	N	L	Q	R	I
LN877755	G	Q	A	I	A	N	R	F	T	S	N	I	K	G	L	T	Q	A	A	R	N	A	N	D	G	I	S	V	A	Q	T	T	E	G	A	L	S	E	I	N	N	N	L	Q	R	I
LN877756	G	Q	A	I	A	N	R	F	T	S	N	I	K	G	L	T	Q	A	A	R	N	A	N	D	G	I	S	V	A	Q	T	T	E	G	A	L	S	E	I	N	N	N	L	Q	R	I
LN877757	G	Q	A	I	A	N	R	F	T	S	N	I	K	G	L	T	Q	A	A	R	N	A	N	D	G	I	S	V	A	Q	T	T	E	G	A	L	S	E	I	N	N	N	L	Q	R	I
LN877758	G	Q	A	I	A	N	R	F	T	S	N	I	K	G	L	T	Q	A	A	R	N	A	N	D	G	I	S	V	A	Q	T	T	E	G	A	L	S	E	I	N	N	N	L	Q	R	I
	G	Q	A	I	A	N	R	F	T	S	N	I	K	G	L	T	Q	A	A	R	N	A	N	D	G	I	S	V	A	Q	T	T	E	G	A	L	S	E	I	N	N	N	L	Q	R	I

Alignment Name: Table S1 AA alignmentUntitled5.aaa_bsml
Length: 595

	Sequence (positions 93–138; markers at 100, 110, 120, 130)
LN877748	R E L T V Q A S T G T N S D S D L D S I Q D E I K S R L D E I D R V S G Q T Q F N G V N V L
LN877749	R E L T V Q A S T G T N S D S D L D S I Q D E I K S R L D E I D R V S G Q T Q F N G V N V L
AY249997	R E L T V Q A S T G T N S D S D L D S I Q D E I K S R L D E I D R V S G Q T Q F N G V N V L
AY337474	R E L T V Q A S T G T N S D S D L D S I Q D E I K S R L D E I D R V S G Q T Q F N G V N V L
AY337471	R E L T V Q A S T G T N S D S D L D S I Q D E I K S R L D E I D R V S G Q T Q F N G V N V L
LN877750	R E L T V Q A S T G T N S D S D L D S I Q D E I K S R L D E I D R V S G Q T Q F N G V N V L
LN877751	R E L T V Q A S T G T N S D S D L D S I Q D E I K S R L D E I D R V S G Q T Q F N G V N V L
LN877752	R E L T V Q A S T G T N S D S D L D S I Q D E I K S R L D E I D R V S G Q T Q F N G V N V L
AB028471	R E L T V Q A S T G T N S D S D L D S I Q D E I K S R L D E I D R V S G Q T Q F N G V N V L
AE014075	R E L T V Q A S T G T N S D S D L D S I Q D E I K S R L D E I D R V S G Q T Q F N G V N V L
CP001671	R E L T V Q A S T G T N S D S D L D S I Q D E I K S R L D E I D R V S G Q T Q F N G V N V L
LN877753	R E L T V Q A S T G T N S D S D L D S I Q D E I K S R L D E I D R V S G Q T Q F N G V N V L
CP009072	R E L T V Q A S T G T N S D S D L D S I Q D E I K S R L D E I D R V S G Q T Q F N G V N V L
CP001855	R E L T V Q A S T G T N S D S D L D S I Q D E I K S R L D E I D R V S G Q T Q F N G V N V L
CU651637	R E L T V Q A S T G T N S D S D L D S I Q D E I K S R L D E I D R V S G Q T Q F N G V N V L
JF308285	R E L T V Q A S T G T N S D S D L D S I Q D E I K S R L D E I D R V S G Q T Q F N G V N V L
LN877754	R E L T V Q A S T G T N S D S D L D S I Q D E I K S R L D E I D R V S G Q T Q F N G V N V L
LN877755	R E L T V Q A S T G T N S D S D L D S I Q D E I K S R L D E I D R V S G Q T Q F N G V N V L
LN877756	R E L T V Q A S T G T N S D S D L D S I Q D E I K S R L D E I D R V S G Q T Q F N G V N V L
LN877757	R E L T V Q A S T G T N S D S D L D S I Q D E I K S R L D E I D R V S G Q T Q F N G V N V L
LN877758	R E L T V Q A S T G T N S D S D L D S I Q D E I K S R L D E I D R V S G Q T Q F N G V N V L
	R E L T V Q A S T G T N S D S D L D S I Q D E I K S R L D E I D R V S G Q T Q F N G V N V L

Alignment Name: Table S1 AA alignmentUntitled5.aaa_bsml
Length: 595

		140										150										160										170										180				
LN877748	A	K	D	G	S	M	K	I	Q	V	G	A	N	D	G	Q	T	I	T	I	D	L	K	K	I	D	S	D	T	L	G	L	N	G	F	N	V	N	G	S	G	T	I	A	N	K
LN877749	A	K	D	G	S	M	K	I	Q	V	G	A	N	D	G	Q	T	I	T	I	D	L	K	K	I	D	S	D	T	L	G	L	N	G	F	N	V	N	G	S	G	T	I	A	N	K
AY249997	A	K	D	G	S	M	K	I	Q	V	G	A	N	D	G	Q	T	I	T	I	D	L	K	K	I	D	S	D	T	L	G	L	N	G	F	N	V	N	G	S	G	T	I	A	N	K
AY337474	A	K	D	G	S	M	K	I	Q	V	G	A	N	D	G	Q	T	I	T	I	D	L	K	K	I	D	S	D	T	L	G	L	N	G	F	N	V	N	G	S	G	T	I	A	N	K
AY337471	A	K	D	G	S	M	K	I	Q	V	G	A	N	D	G	Q	T	I	T	I	D	L	K	K	I	D	S	D	T	L	G	L	N	G	F	N	V	N	G	S	G	T	I	A	N	K
LN877750	A	K	D	G	S	M	K	I	Q	V	G	A	N	D	G	Q	T	I	T	I	D	L	K	K	I	D	S	D	T	L	G	L	N	G	F	N	V	N	G	S	G	T	I	A	N	K
LN877751	A	K	D	G	S	M	K	I	Q	V	G	A	N	D	G	Q	T	I	T	I	D	L	K	K	I	D	S	D	T	L	G	L	N	G	F	N	V	N	G	S	G	T	I	A	N	K
LN877752	A	K	D	G	S	M	K	I	Q	V	G	A	N	D	G	Q	T	I	T	I	D	L	K	K	I	D	S	D	T	L	G	L	N	G	F	N	V	N	G	S	G	T	I	A	N	K
AB028471	A	K	D	G	S	M	K	I	Q	V	G	A	N	D	G	Q	T	I	T	I	D	L	K	K	I	D	S	D	T	L	G	L	N	G	F	N	V	N	G	S	G	T	I	A	N	K
AE014075	A	K	D	G	S	M	K	I	Q	V	G	A	N	D	G	Q	T	I	T	I	D	L	K	K	I	D	S	D	T	L	G	L	N	G	F	N	V	N	G	S	G	T	I	A	N	K
CP001671	A	K	D	G	S	M	K	I	Q	V	G	A	N	D	G	Q	T	I	T	I	D	L	K	K	I	D	S	D	T	L	G	L	N	G	F	N	V	N	G	S	G	T	I	A	N	K
LN877753	A	K	D	G	S	M	K	I	Q	V	G	A	N	D	G	Q	T	I	T	I	D	L	K	K	I	D	S	D	T	L	G	L	N	G	F	N	V	N	G	S	G	T	I	A	N	K
CP009072	A	K	D	G	S	M	K	I	Q	V	G	A	N	D	G	Q	T	I	T	I	D	L	K	K	I	D	S	D	T	L	G	L	N	G	F	N	V	N	G	S	G	T	I	A	N	K
CP001855	A	K	D	G	S	M	K	I	Q	V	G	A	N	D	G	Q	T	I	T	I	D	L	K	K	I	D	S	D	T	L	G	L	N	G	F	N	V	N	G	S	G	T	I	A	N	K
CU651637	A	K	D	G	S	M	K	I	Q	V	G	A	N	D	G	Q	T	I	T	I	D	L	K	K	I	D	S	D	T	L	G	L	N	G	F	N	V	N	G	S	G	T	I	A	N	K
JF308285	A	K	D	G	S	M	K	I	Q	V	G	A	N	D	G	Q	T	I	T	I	D	L	K	K	I	D	S	D	T	L	G	L	N	G	F	N	V	N	G	S	G	T	I	A	N	K
LN877754	A	K	D	G	S	M	K	I	Q	V	G	A	N	D	G	Q	T	I	T	I	D	L	K	K	I	D	S	D	T	L	G	L	N	G	F	N	V	N	G	S	G	T	I	A	N	K
LN877755	A	K	D	G	S	M	K	I	Q	V	G	A	N	D	G	Q	T	I	T	I	D	L	K	K	I	D	S	D	T	L	G	L	N	G	F	N	V	N	G	S	G	T	I	A	N	K
LN877756	A	K	D	G	S	M	K	I	Q	V	G	A	N	D	G	Q	T	I	T	I	D	L	K	K	I	D	S	D	T	L	G	L	N	G	F	N	V	N	G	S	G	T	I	A	N	K
LN877757	A	K	D	G	S	M	K	I	Q	V	G	A	N	D	G	Q	T	I	T	I	D	L	K	K	I	D	S	D	T	L	G	L	N	G	F	N	V	N	G	S	G	T	I	A	N	K
LN877758	A	K	D	G	S	M	K	I	Q	V	G	A	N	D	G	Q	T	I	T	I	D	L	K	K	I	D	S	D	T	L	G	L	N	G	F	N	V	N	G	S	G	T	I	A	N	K
	A	K	D	G	S	M	K	I	Q	V	G	A	N	D	G	Q	T	I	T	I	D	L	K	K	I	D	S	D	T	L	G	L	N	G	F	N	V	N	G	S	G	T	I	A	N	K

Alignment Name: Table S1 AA alignmentUntitled5.aaa_bsml
Length: 595

						190										200										210										220										230
LN877748	A	A	T	I	S	D	L	T	A	A	K	M	D	A	A	T	N	T	I	T	T	T	N	N	A	L	T	A	S	K	A	L	D	Q	L	K	D	G	D	T	V	T	I	K	A	D
LN877749	A	A	T	I	S	D	L	T	A	A	K	M	D	A	A	T	N	T	I	T	T	T	N	N	A	L	T	A	S	K	A	L	D	Q	L	K	D	G	D	T	V	T	I	K	A	D
AY249997	A	A	T	I	S	D	L	T	A	A	K	M	D	A	A	T	N	T	I	T	T	T	N	N	A	L	T	A	S	K	A	L	D	Q	L	K	D	G	D	T	V	T	I	K	A	D
AY337474	A	A	T	I	S	D	L	T	A	A	K	M	D	A	A	T	N	T	I	T	T	T	N	N	A	L	T	A	S	K	A	L	D	Q	L	K	D	G	D	T	V	T	I	K	A	D
AY337471	A	A	T	I	S	D	L	T	A	A	K	M	D	A	A	T	N	T	I	T	T	T	N	N	A	L	T	A	S	K	A	L	D	Q	L	K	D	G	D	T	V	T	I	K	A	D
LN877750	A	A	T	I	S	D	L	T	A	A	K	M	D	A	A	T	N	T	I	T	T	T	N	N	A	L	T	A	S	K	A	L	D	Q	L	K	D	G	D	T	V	T	I	K	A	D
LN877751	A	A	T	I	S	D	L	T	A	A	K	M	D	A	A	T	N	T	I	T	T	T	N	N	A	L	T	A	S	K	A	L	D	Q	L	K	D	G	D	T	V	T	I	K	A	D
LN877752	A	A	T	I	S	D	L	T	A	A	K	M	D	A	A	T	N	T	I	T	T	T	N	N	A	L	T	A	S	K	A	L	D	Q	L	K	D	G	D	T	V	T	I	K	A	D
AB028471	A	A	T	I	S	D	L	T	A	A	K	M	D	A	A	T	N	T	I	T	T	T	N	N	A	L	T	A	S	K	A	L	D	Q	L	K	D	G	D	T	V	T	I	K	A	D
AE014075	A	A	T	I	S	D	L	T	A	A	K	M	D	A	A	T	N	T	I	T	T	T	N	N	A	L	T	A	S	K	A	L	D	Q	L	K	D	G	D	T	V	T	I	K	A	D
CP001671	A	A	T	I	S	D	L	T	A	A	K	M	D	A	A	T	N	T	I	T	T	T	N	N	A	L	T	A	S	K	A	L	D	Q	L	K	D	G	D	T	V	T	I	K	A	D
LN877753	A	A	T	I	S	D	L	T	A	A	K	M	D	A	A	T	N	T	I	T	T	T	N	N	A	L	T	A	S	K	A	L	D	Q	L	K	D	G	D	T	V	T	I	K	A	D
CP009072	A	A	T	I	S	D	L	T	A	A	K	M	D	A	A	T	N	T	I	T	T	T	N	N	A	L	T	A	S	K	A	L	D	Q	L	K	D	G	D	T	V	T	I	K	A	D
CP001855	A	A	T	I	S	D	L	T	A	A	K	M	D	A	A	T	N	T	I	T	T	T	N	N	A	L	T	A	S	K	A	L	D	Q	L	K	D	G	D	T	V	T	I	K	A	D
CU651637	A	A	T	I	S	D	L	T	A	A	K	M	D	A	A	T	N	T	I	T	T	T	N	N	A	L	T	A	S	K	A	L	D	Q	L	K	D	G	D	T	V	T	I	K	A	D
JF308285	A	A	T	I	S	D	L	T	A	A	K	M	D	A	A	T	N	T	I	T	T	T	N	N	A	L	T	A	S	K	A	L	D	Q	L	K	D	G	D	T	V	T	I	K	A	D
LN877754	A	A	T	I	S	D	L	T	A	A	K	M	D	A	A	T	N	T	I	T	T	T	N	N	A	L	T	A	S	K	A	L	D	Q	L	K	D	G	D	T	V	T	I	K	A	D
LN877755	A	A	T	I	S	D	L	T	A	A	K	M	D	A	A	T	N	T	I	T	T	T	N	N	A	L	T	A	S	K	A	L	D	Q	L	K	D	G	D	T	V	T	I	K	A	D
LN877756	A	A	T	I	S	D	L	T	A	A	K	M	D	A	A	T	N	T	I	T	T	T	N	N	A	L	T	A	S	K	A	L	D	Q	L	K	D	G	D	T	V	T	I	K	A	D
LN877757	A	A	T	I	S	D	L	T	A	A	K	M	D	A	A	T	N	T	I	T	T	T	N	N	A	L	T	A	S	K	A	L	D	Q	L	K	D	G	D	T	V	T	I	K	A	D
LN877758	A	A	T	I	S	D	L	T	A	A	K	M	D	A	A	T	N	T	I	T	T	T	N	N	A	L	T	A	S	K	A	L	D	Q	L	K	D	G	D	T	V	T	I	K	A	D
	A	A	T	I	S	D	L	T	A	A	K	M	D	A	A	T	N	T	I	T	T	T	N	N	A	L	T	A	S	K	A	L	D	Q	L	K	D	G	D	T	V	T	I	K	A	D

Alignment Name: Table S1 AA alignmentUntitled5.aaa_bsml
Length: 595

Ruler positions: 240 (column 10), 250 (column 20), 260 (column 30), 270 (column 40)

LN877748	A A Q T A T V Y T Y N A S A G N F S F S N V S N N T S K K A G D V A A S L L P P A G Q T A S
LN877749	A A Q T A T V Y T Y N A S A G N F S F S N V S N N T S A K A G D V A A S L L P P A G Q T A S
AY249997	A A Q T A T V Y T Y N A S A G N F S F S N V S N N T S A K A G D V A A S L L P P A G Q T A S
AY337474	A A Q T A T V Y T Y N A S A G N F S L S N V S N N T S E K A G D V A A S L L P P A G Q T A S
AY337471	A A Q T A T V Y T Y N A S A G N F S F S N V S N N T S E K A G D V A A S L L P P A G Q T A S
LN877750	A A Q T A T V Y T Y N A S A G N F S F S N V S N N T S E K A G D V A A S L L P P A G Q T A S
LN877751	A A Q T A T V Y T Y N A S A G N F S F S N V S N N T S E K A G D V A A S L L P P A G Q T A S
LN877752	A A Q T A T V Y T Y N A S A G N F S F S N V S N N T S E K A G D V A A S L L P P A G Q T A S
AB028471	A A Q T A T V Y T Y N A S A G N F S F S N V S N N T S A K A G D V A A S L L P P A G Q T A S
AE014075	A A Q T A T V Y T Y N A S A G N F S F S N V S N N T S A K A G D V A A S L L P P A G Q T A S
CP001671	A A Q T A T V Y T Y N A S A G N F S F S N V S N N T S A K A G D V A A S L L P P A G Q T A S
LN877753	A A Q T A T V Y T Y N A S A G N F S F S N V S N N T S A K A G D V A A S L L P P A G Q T A S
CP009072	A A Q T A T V Y T Y N A S A G N F S F S N V S N N T S A K A G D V A A S L L P P A G Q T A S
CP001855	A A Q T A T V Y T Y N A S A G N F S F S N V S N N T S A K A G D V A A S L L P P A G Q T A S
CU651637	A A Q T A T V Y T Y N A S A G N F S F S N V S N N T S A K A G D V A A S L L P P A G Q T A S
JF308285	A A Q T A T V Y T Y N A S A G N F S F S N V S N N T S A K A G D V A A S L L P P A G Q T A S
LN877754	A A Q T A T V Y T Y N A S A G N F S F S N V S N N T S T K A G D V A A S L L P P A G Q T A S
LN877755	A A Q T A T V Y T Y N A S A G N F S F S N V S N N T S T K A G D V A A S L L P P A G Q T A S
LN877756	A A Q T A T V Y T Y N A S A G N F S F S N V S N N T S T K A G D V A A S L L P P A G Q T A S
LN877757	A A Q T A T V Y T Y N A S A G N F S F S N V S N N T S T K A G D V A A S L L P P A G Q T A S
LN877758	A A Q T A T V Y T Y N A S A G N F S F S N V S N N T S T K A G D V A A S L L P P A G Q T A S
	A A Q T A T V Y T Y N A S A G N F S f S N V S N N T S X K A G D V A A S L L P P A G Q T A S

Alignment Name: Table S1 AA alignmentUntitled5.aaa_bsml
Length: 595

	280 · 290 · 300 · 310 · 320
LN877748	GVYKAASGEVNFDVDANGKITIGGQKAYLTSDGNLTTNDAGGATAA
LN877749	GVYKAASGEVNFDVDANGKITIGGQKAYLTSDGNLTTNDAGGATAA
AY249997	GVYKAASGEVNFDVDANGKITIGGQKAYLTSDGNLTTNDAGGATAA
AY337474	GVYKAASGEVNFDVDANGKITIGGQKAYLTSDGNLTTNDAGGATAA
AY337471	GVYKAASGEVNFDVDANGKITIGGQKAYLTSDGNLTTNDAGGATAA
LN877750	GVYKAASGEVNFDVDANGKITIGGQKAYLTSDGNLTTNDAGGATAA
LN877751	GVYKAASGEVNFDVDANGKITIGGQKAYLTSDGNLTTNDAGGATAA
LN877752	GVYKAASGEVNFDVDANGKITIGGQKAYLTSDGNLTTNDAGGATAA
AB028471	GVYKAASGEVNFDVDANGKITIGGQEAYLTSDGNLTTNDAGGATAA
AE014075	GVYKAASGEVNFDVDANGKITIGGQEAYLTSDGNLTTNDAGGATAA
CP001671	GVYKAASGEVNFDVDANGKITIGGQEAYLTSDGNLTTNDAGGATAA
LN877753	GVYKAASGEVNFDVDANGKITIGGQEAYLTSDGNLTTNDAGGATAA
CP009072	GVYKAASGEVNFDVDANGKITIGGQEAYLTSDGNLTTNDAGGATAA
CP001855	GVYKAASGEVNFDVDANGKITIGGQEAYLTSDGNLTTNDAGGATAA
CU651637	GVYKAASGEVNFDVDANGKITIGGQEAYLTSDGNLTTNDAGGATAA
JF308285	GVYKAASGEVNFDVDANGKITIGGQEAYLTSDGNLTTNDAGGATAA
LN877754	GVYKAASGEVNFDVDANGKITIGGQEAYLTSDGNLTTNDAGGATAA
LN877755	GVYKAASGEVNFDVDANGKITIGGQEAYLTSDGNLTTNDAGGATAA
LN877756	GVYKAASGEVNFDVDANGKITIGGQEAYLTSDGNLTTNDAGGATAA
LN877757	GVYKAASGEVNFDVDANGKITIGGQEAYLTSDGNLTTNDAGGATAA
LN877758	GVYKAASGEVNFDVDANGKITIGGQEAYLTSDGNLTTNDAGGATAA
	GVYKAASGEVNFDVDANGKITIGGQeAYLTSDGNLTTNDAGGATAA

Alignment Name: Table S1 AA alignmentUntitled5.aaa_bsml
Length: 595

	330 ·········· 340 ·········· 350 ·········· 360
LN877748	TLDGLFKKAGDGQSIGFKKTASVTMGGTTYNFKTGADADAATANAG
LN877749	TLDGLFKKAGDGQSIGFKKTASVTMGGTTYNFKTGADADAATANAG
AY249997	TLDGLFKKAGDGQSIGFKKTASVTMGGTTYNFKTGADADAATANAG
AY337474	TLDGLFKKAGDGQSIGFKKTASVTMGGTTYNFKTGADADAATANAG
AY337471	TLDGLFKKAGDGQSIGFKKTASVTMGGTTYNFKTGADADAATANAG
LN877750	TLDGLFKKAGDGQSIGFKKTASVTMGGTTYNFKTGADADAATANAG
LN877751	TLDGLFKKAGDGQSIGVKKTASVTMGGTTYNFKTGADADAATANAG
LN877752	TLDGLFKKAGDGQSIGVKKTASVTMGGTTYNFKTGADADAATANAG
AB028471	TLDGLFKKAGDGQSIGFNKTASVTMGGTTYNFKTGADAGAATANAG
AE014075	TLDGLFKKAGDGQSIGFNKTASVTMGGTTYNFKTGADAGAATANAG
CP001671	TLDGLFKKAGDGQSIGFNKTASVTMGGTTYNFKTGADAGAATANAG
LN877753	TLDGLFKKAGDGQSIGFNKTASVTMGGTTYNFKTGADAGAATANAG
CP009072	TLDGLFKKAGDGQSIGFNKTASVTMGGTTYNFKTGADAGAATANAG
CP001855	TLDGLFKKAGDGQSIGFNKTASVTMGGTTYNFKTGADAGAATANAG
CU651637	TLDGLFKKAGDGQSIGFNKTASVTMGGTTYNFKTGADAGAATANAG
JF308285	TLDGLFKKAGDGQSIGFNKTASVTMGGTTYNFKTGADAGAATANAG
LN877754	TLDGLFKKAGDGQSIGFNKTASVTMGGTTYNFKTGADAGAATANAG
LN877755	TLDGLFKKAGDGQSIGFNKTASVTMGGTTYNFKTGADAGAATANAG
LN877756	TLDGLFKKAGDGQSIGFNKTASVTMGGTTYNFKTGADAGAATANAG
LN877757	TLDGLFKKAGDGQSIGFNKTASVTMGGTTYNFKTGADAGAATANAG
LN877758	TLDGLFKKAGDGQSIGFNKTASVTMGGTTYNFKTGADAGAATANAG
	TLDGLFKKAGDGQSIGfnKTASVTMGGTTYNFKTGADAgAATANAG

Alignment Name: Table S1 AA alignmentUntitled5.aaa_bsml
Length: 595

		370										380										390										400										410				
LN877748	V	S	F	T	D	T	A	S	K	E	T	V	L	N	K	V	A	T	A	K	Q	G	K	A	A	A	A	D	G	D	T	S	A	T	I	T	Y	K	S	G	V	Q	T	Y	Q	A
LN877749	V	S	F	T	D	T	A	S	K	E	T	V	L	N	K	V	A	T	A	K	Q	G	K	A	V	A	A	D	G	D	T	S	A	T	I	T	Y	K	S	G	V	Q	T	Y	Q	A
AY249997	V	S	F	T	D	T	A	S	K	E	T	V	L	N	K	V	A	T	A	K	Q	G	K	A	V	A	A	D	G	D	T	S	A	T	I	T	Y	K	S	G	V	Q	T	Y	Q	A
AY337474	V	S	F	T	D	T	A	S	K	E	T	V	L	N	K	V	A	T	A	K	Q	G	K	A	A	A	A	D	G	D	T	S	A	T	I	T	Y	K	S	G	V	Q	T	Y	Q	A
AY337471	V	S	F	T	D	T	A	S	K	E	T	V	L	N	K	V	A	T	A	K	Q	G	K	A	A	A	A	D	G	D	T	S	A	T	I	T	Y	K	S	G	V	Q	T	Y	Q	A
LN877750	V	S	F	T	D	T	A	S	K	E	T	V	L	N	K	V	A	T	A	K	Q	G	K	A	A	A	A	D	G	D	T	S	A	T	I	T	Y	K	S	G	V	Q	T	Y	Q	A
LN877751	V	S	F	T	D	T	A	S	K	E	T	V	L	N	K	V	A	T	A	K	Q	G	K	A	A	A	A	D	G	D	T	S	A	T	I	T	Y	K	S	G	V	Q	T	Y	Q	A
LN877752	V	S	F	T	D	T	A	S	K	E	T	V	L	N	K	V	A	T	A	K	Q	G	K	A	A	A	A	D	G	D	T	S	A	T	I	T	Y	K	S	G	V	Q	T	Y	Q	A
AB028471	V	S	F	T	D	T	A	S	K	E	T	V	L	N	K	V	A	T	A	K	Q	G	T	A	V	A	A	N	G	D	T	S	A	T	I	T	Y	K	S	G	V	Q	T	Y	Q	A
AE014075	V	S	F	T	D	T	A	S	K	E	T	V	L	N	K	V	A	T	A	K	Q	G	T	A	V	A	A	N	G	D	T	S	A	T	I	T	Y	K	S	G	V	Q	T	Y	Q	A
CP001671	V	S	F	T	D	T	A	S	K	E	T	V	L	N	K	V	A	T	A	K	Q	G	T	A	V	A	A	N	G	D	T	S	A	T	I	T	Y	K	S	G	V	Q	T	Y	Q	A
LN877753	V	S	F	T	D	T	A	S	K	E	T	V	L	N	K	V	A	T	A	K	Q	G	T	A	V	A	A	N	G	D	T	S	A	T	I	T	Y	K	S	G	V	Q	T	Y	Q	A
CP009072	V	S	F	T	D	T	A	S	K	E	T	V	L	N	K	V	A	T	A	K	Q	G	T	A	V	A	A	N	G	D	T	S	A	T	I	T	Y	K	S	G	V	Q	T	Y	Q	A
CP001855	V	S	F	T	D	T	A	S	K	E	T	V	L	N	K	V	A	T	A	K	Q	G	T	A	V	A	A	N	G	D	T	S	A	T	I	T	Y	K	S	G	V	Q	T	Y	Q	A
CU651637	V	S	F	T	D	T	A	S	K	E	T	V	L	N	K	V	A	T	A	K	Q	G	T	A	V	A	A	N	G	D	T	S	A	T	I	T	Y	K	S	G	V	Q	T	Y	Q	A
JF308285	V	S	F	T	D	T	A	S	K	E	T	V	L	N	K	V	A	T	A	K	Q	G	T	A	V	A	A	N	G	D	T	S	A	T	I	T	Y	K	S	G	V	Q	T	Y	Q	A
LN877754	V	S	F	T	D	T	A	S	K	E	T	V	L	N	K	V	A	T	A	K	Q	G	T	A	A	A	A	N	G	D	T	S	A	T	I	T	Y	K	S	G	V	Q	T	Y	Q	A
LN877755	V	S	F	T	D	T	A	S	K	E	T	V	L	N	K	V	A	T	A	K	Q	G	T	A	A	A	A	N	G	D	T	S	A	T	I	T	Y	K	S	G	V	Q	T	Y	Q	A
LN877756	V	S	F	T	D	T	A	S	K	E	T	V	L	N	K	V	A	T	A	K	Q	G	T	A	A	A	A	N	G	D	T	S	A	T	I	T	Y	K	S	G	V	Q	T	Y	Q	A
LN877757	V	S	F	T	D	T	A	S	K	E	T	V	L	N	K	V	A	T	A	K	Q	G	T	A	A	A	A	N	G	D	T	S	A	T	I	T	Y	K	S	G	V	Q	T	Y	Q	A
LN877758	V	S	F	T	D	T	A	S	K	E	T	V	L	N	K	V	A	T	A	K	Q	G	T	A	A	A	A	N	G	D	T	S	A	T	I	T	Y	K	S	G	V	Q	T	Y	Q	A
	V	S	F	T	D	T	A	S	K	E	T	V	L	N	K	V	A	T	A	K	Q	G	t	A	a	A	A	n	G	D	T	S	A	T	I	T	Y	K	S	G	V	Q	T	Y	Q	A

Alignment Name: Table S1 AA alignmentUntitled5.aaa_bsml
Length: 595

	420 430 440 450 460
LN877748	VFAAGDGTASAKYADKADVSNATATYTDADGEMTTIGSYTTKYSID
LN877749	VFAAGDGTASAKYADKADVSNATATYTDADGEMTTIGSYTTKYSID
AY249997	VFAAGDGTASAKYADKADVSNATATYTDADGEMTTIGSYTTKYSID
AY337474	VFAAGDGTASAKYADKADVSNATATYTDADGEMTTIGSYTTKYSID
AY337471	VFAAGDGTASAKYADKADVSNATATYTDADGEMTTIGSYTTKYSID
LN877750	VFAAGDGTASAKYADKADVSNATATYTDADGEMTTIGSYTTKYSID
LN877751	VFAAGDGTASAKYADKADVSNATATYTDADGEMTTIGSYTTKYSID
LN877752	VFAAGDGTASAKYADKADVSNATATYTDADGEMTTIGSYTTKYSID
AB028471	VFAAGDGTASAKYADNTDVSNATATYTDADGEMTTIGSYTTKYSID
AE014075	VFAAGDGTASAKYADNTDVSNATATYTDADGEMTTIGSYTTKYSID
CP001671	VFAAGDGTASAKYADNTDVSNATATYTDADGEMTTIGSYTTKYSID
LN877753	VFAAGDGTASAKYADNTDVSNATATYTDADGEMTTIGSYTTKYSID
CP009072	VFAAGDGTASAKYADNTDVSNATATYTDADGEMTTIGSYTTKYSID
CP001855	VFAAGDGTASAKYADNTDVSNATATYTDADGEMTTIGSYTTKYSID
CU651637	VFAAGDGTASAKYADNTDVSNATATYTDADGEMTTIGSYTTKYSID
JF308285	VFAAGDGTASAKYADNTDVSNATATYTDADGEMTTIGSYTTKYSID
LN877754	VFAAGDGTASAKYADNADVSNATATYTDADGEMTTIGSYTTKYSID
LN877755	VFAAGDGTASAKYADNADVSNATATYTDADGEMTTIGSYTTKYSID
LN877756	VFAAGDGTASAKYADNADVSNATATYTDADGEMTTIGSYTTKYSID
LN877757	VFAAGDGTASAKYADNADVSNATATYTDADGEMTTIGSYTTKYSID
LN877758	VFAAGDGTASAKYADNADVSNATATYTDADGEMTTIGSYTTKYSID
	VFAAGDGTASAKYADnaDVSNATATYTDADGEMTTIGSYTTKYSID

Alignment Name: Table S1 AA alignmentUntitled5.aaa_bsml
Length: 595

										470										480										490										500						
LN877748	A	N	N	G	K	V	T	V	D	S	G	I	G	T	G	K	Y	A	P	K	V	G	A	E	V	Y	V	S	A	N	G	T	L	T	T	D	A	T	S	E	G	T	V	T	K	D
LN877749	A	N	N	G	K	V	T	V	D	S	G	T	G	T	G	K	Y	A	P	K	V	G	A	E	V	Y	V	S	A	N	G	T	L	T	T	D	A	T	S	E	G	T	V	T	K	D
AY249997	A	N	N	G	K	V	T	V	D	S	G	T	G	T	G	K	Y	A	P	K	V	G	A	E	V	Y	V	S	A	N	G	T	L	T	T	D	A	T	S	E	G	T	V	T	K	D
AY337474	A	N	N	G	K	V	T	V	D	S	G	T	G	T	G	K	Y	A	P	K	V	G	A	E	V	Y	V	S	A	N	G	T	L	T	T	D	A	T	S	E	G	T	V	T	K	D
AY337471	A	N	N	G	K	V	T	V	D	S	G	T	G	T	G	K	Y	A	P	K	V	G	A	E	V	Y	V	S	A	N	G	T	L	T	T	D	A	T	S	E	G	T	V	T	K	D
LN877750	A	N	N	G	K	V	T	V	D	S	G	T	G	T	G	K	Y	A	P	K	V	G	A	E	V	Y	V	S	A	N	G	T	L	T	T	D	A	T	S	E	G	T	V	T	K	D
LN877751	A	N	N	G	K	V	T	V	D	S	G	T	G	T	G	K	Y	A	P	K	V	G	A	E	V	Y	V	S	A	N	G	T	L	T	T	D	A	T	S	E	G	T	V	T	K	D
LN877752	A	N	N	G	K	V	T	V	D	S	G	T	G	T	G	K	Y	A	P	K	V	G	A	E	V	Y	V	S	A	N	G	T	L	T	T	D	A	T	S	E	G	T	V	T	K	D
AB028471	A	N	N	G	K	V	T	V	D	S	G	T	G	T	G	K	Y	A	P	K	V	G	A	E	V	Y	V	S	A	N	G	T	L	T	T	D	A	T	S	E	G	T	V	T	K	D
AE014075	A	N	N	G	K	V	T	V	D	S	G	T	G	T	G	K	Y	A	P	K	V	G	A	E	V	Y	V	S	A	N	G	T	L	T	T	D	A	T	S	E	G	T	V	T	K	D
CP001671	A	N	N	G	K	V	T	V	D	S	G	T	G	T	G	K	Y	A	P	K	V	G	A	E	V	Y	V	S	A	N	G	T	L	T	T	D	A	T	S	E	G	T	V	T	K	D
LN877753	A	N	N	G	K	V	T	V	D	S	G	T	G	T	G	K	Y	A	P	K	V	G	A	E	V	Y	V	S	A	N	G	T	L	T	T	D	A	T	S	E	G	T	V	T	K	D
CP009072	A	N	N	G	K	V	T	V	D	S	G	T	G	T	G	K	Y	A	P	K	V	G	A	E	V	Y	V	S	A	N	G	T	L	T	T	D	A	T	S	E	G	T	V	T	K	D
CP001855	A	N	N	G	K	V	T	V	D	S	G	T	G	T	G	K	Y	A	P	K	V	G	A	E	V	Y	V	S	A	N	G	T	L	T	T	D	A	T	S	E	G	T	V	T	K	D
CU651637	A	N	N	G	K	V	T	V	D	S	G	T	G	T	G	K	Y	A	P	K	V	G	A	E	V	Y	V	S	A	N	G	T	L	T	T	D	A	T	S	E	G	T	V	T	K	D
JF308285	A	N	N	G	K	V	T	V	D	S	G	T	G	T	G	K	Y	A	P	K	V	G	A	E	V	Y	V	S	A	N	G	T	L	T	T	D	A	T	S	E	G	T	V	T	K	D
LN877754	A	N	N	G	K	V	T	V	D	S	G	T	G	T	G	K	Y	A	P	K	V	G	A	E	V	Y	V	S	A	N	G	T	L	T	T	D	A	T	S	E	G	T	V	T	K	D
LN877755	A	N	N	G	K	V	T	V	D	S	G	T	G	T	G	K	Y	A	P	K	V	G	A	E	V	Y	V	S	A	N	G	T	L	T	T	D	A	T	S	E	G	T	V	T	K	D
LN877756	A	N	N	G	K	V	T	V	D	S	G	T	G	T	G	K	Y	A	P	K	I	G	A	E	V	Y	V	S	A	N	G	T	L	T	T	D	A	T	S	E	G	T	V	T	K	D
LN877757	A	N	N	G	K	V	T	V	D	S	G	T	G	T	G	K	Y	A	P	K	I	G	A	E	V	Y	V	S	A	N	G	T	L	T	T	D	A	T	S	E	G	T	V	T	K	D
LN877758	A	N	N	G	K	V	T	V	D	S	G	T	G	T	G	K	Y	A	P	K	I	G	A	E	V	Y	V	S	A	N	G	T	L	T	T	D	A	T	S	E	G	T	V	T	K	D
	A	N	N	G	K	V	T	V	D	S	G	t	G	T	G	K	Y	A	P	K	v	G	A	E	V	Y	V	S	A	N	G	T	L	T	T	D	A	T	S	E	G	T	V	T	K	D

Alignment Name: Table S1 AA alignmentUntitled5.aaa_bsml
Length: 595

				510										520										530										540										550		
LN877748	P	L	K	A	L	D	E	A	I	S	S	I	D	K	F	R	S	S	L	G	A	I	Q	N	R	L	D	S	A	V	T	N	L	N	N	T	T	T	N	L	S	E	A	Q	S	R
LN877749	P	L	K	A	L	D	E	A	I	S	S	I	D	K	F	R	S	S	L	G	A	I	Q	N	R	L	D	S	A	V	T	N	L	N	N	T	T	T	N	L	S	E	A	Q	S	R
AY249997	P	L	K	A	L	D	E	A	I	S	S	I	D	K	F	R	S	S	L	G	A	I	Q	N	R	L	D	S	A	V	T	N	L	N	N	T	T	T	N	L	S	E	A	Q	S	R
AY337474	P	L	K	A	L	D	E	A	I	S	S	I	D	K	F	R	S	S	L	G	A	I	Q	N	R	L	D	S	A	V	T	N	L	N	N	T	T	T	N	L	S	E	A	Q	S	R
AY337471	P	L	K	A	L	D	E	A	I	S	S	I	D	K	F	R	S	S	L	G	A	I	Q	N	R	L	D	S	A	V	T	N	L	N	N	T	T	T	N	L	S	E	A	Q	S	R
LN877750	P	L	K	A	L	D	E	A	I	S	S	I	D	K	F	R	S	S	L	G	A	I	Q	N	R	L	D	S	A	V	T	N	L	N	N	T	T	T	N	L	S	E	A	Q	S	R
LN877751	P	L	K	A	L	D	E	A	I	S	S	I	D	K	F	R	S	S	L	G	A	I	Q	N	R	L	D	S	A	V	T	N	L	N	N	T	T	T	N	L	S	E	A	Q	S	R
LN877752	P	L	K	A	L	D	E	A	I	S	S	I	D	K	F	R	S	S	L	G	A	I	Q	N	R	L	D	S	A	V	T	N	L	N	N	T	T	T	N	L	S	E	A	Q	S	R
AB028471	P	L	K	A	L	D	E	A	I	S	S	I	D	K	F	R	S	S	L	G	A	I	Q	N	R	L	D	S	A	V	T	N	L	N	N	T	T	T	N	L	S	E	A	Q	S	R
AE014075	P	L	K	A	L	D	E	A	I	S	S	I	D	K	F	R	S	S	L	G	A	I	Q	N	R	L	D	S	A	V	T	N	L	N	N	T	T	T	N	L	S	E	A	Q	S	R
CP001671	P	L	K	A	L	D	E	A	I	S	S	I	D	K	F	R	S	S	L	G	A	I	Q	N	R	L	D	S	A	V	T	N	L	N	N	T	T	T	N	L	S	E	A	Q	S	R
LN877753	P	L	K	A	L	D	E	A	I	S	S	I	D	K	F	R	S	S	L	G	A	I	Q	N	R	L	D	S	A	V	T	N	L	N	N	T	T	T	N	L	S	E	A	Q	S	R
CP009072	P	L	K	A	L	D	E	A	I	S	S	I	D	K	F	R	S	S	L	G	A	I	Q	N	R	L	D	S	A	V	T	N	L	N	N	T	T	T	N	L	S	E	A	Q	S	R
CP001855	P	L	K	A	L	D	E	A	I	S	S	I	D	K	F	R	S	S	L	G	A	I	Q	N	R	L	D	S	A	V	T	N	L	N	N	T	T	T	N	L	S	E	A	Q	S	R
CU651637	P	L	K	A	L	D	E	A	I	S	S	I	D	K	F	R	S	S	L	G	A	I	Q	N	R	L	D	S	A	V	T	N	L	N	N	T	T	T	N	L	S	E	A	Q	S	R
JF308285	P	L	K	A	L	D	E	A	I	S	S	I	D	K	F	R	S	S	L	G	A	I	Q	N	R	L	D	S	A	V	T	N	L	N	N	T	T	T	N	L	S	E	A	Q	S	R
LN877754	P	L	K	A	L	D	E	A	I	S	S	I	D	K	F	R	S	S	L	G	A	I	Q	N	R	L	D	S	A	V	T	N	L	N	N	T	T	T	N	L	S	E	A	Q	S	R
LN877755	P	L	K	A	L	D	E	A	I	S	S	I	D	K	F	R	S	S	L	G	A	I	Q	N	R	L	D	S	A	V	T	N	L	N	N	T	T	T	N	L	S	E	A	Q	S	R
LN877756	P	L	K	A	L	D	E	A	I	S	S	I	D	K	F	R	S	S	L	G	A	I	Q	N	R	L	D	S	A	V	T	N	L	N	N	T	T	T	N	L	S	E	A	Q	S	R
LN877757	P	L	K	A	L	D	E	A	I	S	S	I	D	K	F	R	S	S	L	G	A	I	Q	N	R	L	D	S	A	V	T	N	L	N	N	T	T	T	N	L	S	E	A	Q	S	R
LN877758	P	L	K	A	L	D	E	A	I	S	S	I	D	K	F	R	S	S	L	G	A	I	Q	N	R	L	D	S	A	V	T	N	L	N	N	T	T	T	N	L	S	E	A	Q	S	R
	P	L	K	A	L	D	E	A	I	S	S	I	D	K	F	R	S	S	L	G	A	I	Q	N	R	L	D	S	A	V	T	N	L	N	N	T	T	T	N	L	S	E	A	Q	S	R

Alignment Name: Table S1 AA alignmentUntitled5.aaa_bsml
Length: 595

								560										570										580										590					
LN877748	I	Q	D	A	D	Y	A	T	E	V	S	N	M	S	K	A	Q	I	I	Q	Q	A	G	N	S	V	L	A	K	A	N	Q	V	P	Q	Q	V	L	S	L	L	Q	G
LN877749	I	Q	D	A	D	Y	A	T	E	V	S	N	M	S	K	A	Q	I	I	Q	Q	A	G	N	S	V	L	A	K	A	N	Q	V	P	Q	Q	V	L	S	L	L	Q	G
AY249997	I	Q	D	A	D	Y	A	T	E	V	S	N	M	S	K	A	Q	I	I	Q	Q	A	G	N	S	V	L	A	K	A	N	Q	V	P	Q	Q	V	L	S	L	L	Q	G
AY337474	I	Q	D	A	D	Y	A	T	E	V	S	N	M	S	K	A	Q	I	I	Q	Q	A	G	N	S	V	L	A	K	A	N	Q	V	P	Q	Q	V	L	S	L	L	Q	G
AY337471	I	Q	D	A	D	Y	A	T	E	V	S	N	M	S	K	A	Q	I	I	Q	Q	A	G	N	S	V	L	A	K	A	N	Q	V	P	Q	Q	V	L	S	L	L	Q	G
LN877750	I	Q	D	A	D	Y	A	T	E	V	S	N	M	S	K	A	Q	I	I	Q	Q	A	G	N	S	V	L	A	K	A	N	Q	V	P	Q	Q	V	L	S	L	L	Q	G
LN877751	I	Q	D	A	D	Y	A	T	E	V	S	N	M	S	K	A	Q	I	I	Q	Q	A	G	N	S	V	L	A	K	A	N	Q	V	P	Q	Q	V	L	S	L	L	Q	G
LN877752	I	Q	D	A	D	Y	A	T	E	V	S	N	M	S	K	A	Q	I	I	Q	Q	A	G	N	S	V	L	A	K	A	N	Q	V	P	Q	Q	V	L	S	L	L	Q	G
AB028471	I	Q	D	A	D	Y	A	T	E	V	S	N	M	S	K	A	Q	I	I	Q	Q	A	G	N	S	V	L	A	K	A	N	Q	V	P	Q	Q	V	L	S	L	L	Q	G
AE014075	I	Q	D	A	D	Y	A	T	E	V	S	N	M	S	K	A	Q	I	I	Q	Q	A	G	N	S	V	L	A	K	A	N	Q	V	P	Q	Q	V	L	S	L	L	Q	G
CP001671	I	Q	D	A	D	Y	A	T	E	V	S	N	M	S	K	A	Q	I	I	Q	Q	A	G	N	S	V	L	A	K	A	N	Q	V	P	Q	Q	V	L	S	L	L	Q	G
LN877753	I	Q	D	A	D	Y	A	T	E	V	S	N	M	S	K	A	Q	I	I	Q	Q	A	G	N	S	V	L	A	K	A	N	Q	V	P	Q	Q	V	L	S	L	L	Q	G
CP009072	I	Q	D	A	D	Y	A	T	E	V	S	N	M	S	K	A	Q	I	I	Q	Q	A	G	N	S	V	L	A	K	A	N	Q	V	P	Q	Q	V	L	S	L	L	Q	G
CP001855	I	Q	D	A	D	Y	A	T	E	V	S	N	M	S	K	A	Q	I	I	Q	Q	A	G	N	S	V	L	A	K	A	N	Q	V	P	Q	Q	V	L	S	L	L	Q	G
CU651637	I	Q	D	A	D	Y	A	T	E	V	S	N	M	S	K	A	Q	I	I	Q	Q	A	G	N	S	V	L	A	K	A	N	Q	V	P	Q	Q	V	L	S	L	L	Q	G
JF308285	I	Q	D	A	D	Y	A	T	E	V	S	N	M	S	K	A	Q	I	I	Q	Q	A	G	N	S	V	L	A	K	A	N	Q	V	P	Q	Q	V	L	S	L	L	Q	G
LN877754	I	Q	D	A	D	Y	A	T	E	V	S	N	M	S	K	A	Q	I	I	Q	Q	A	G	N	S	V	L	A	K	A	N	Q	V	P	Q	Q	V	L	S	L	L	Q	G
LN877755	I	Q	D	A	D	Y	A	T	E	V	S	N	M	S	K	A	Q	I	I	Q	Q	A	G	N	S	V	L	A	K	A	N	Q	V	P	Q	Q	V	L	S	L	L	Q	G
LN877756	I	Q	D	A	D	Y	A	T	E	V	S	N	M	S	K	A	Q	I	I	Q	Q	A	G	N	S	V	L	A	K	A	N	Q	V	P	Q	Q	V	L	S	L	L	Q	G
LN877757	I	Q	D	A	D	Y	A	T	E	V	S	N	M	S	K	A	Q	I	I	Q	Q	A	G	N	S	V	L	A	K	A	N	Q	V	P	Q	Q	V	L	S	L	L	Q	G
LN877758	I	Q	D	A	D	Y	A	T	E	V	S	N	M	S	K	A	Q	I	I	Q	Q	A	G	N	S	V	L	A	K	A	N	Q	V	P	Q	Q	V	L	S	L	L	Q	G
	I	Q	D	A	D	Y	A	T	E	V	S	N	M	S	K	A	Q	I	I	Q	Q	A	G	N	S	V	L	A	K	A	N	Q	V	P	Q	Q	V	L	S	L	L	Q	G
